# Contemporary epidemiology of rising atrial septal defect trends across USA 1991–2016: a combined ecological geospatiotemporal and causal inferential study

**DOI:** 10.1186/s12887-020-02431-z

**Published:** 2020-11-30

**Authors:** Albert Stuart Reece, Gary Kenneth Hulse

**Affiliations:** 1 Division of Psychiatry, University of Western Australia, Crawley, Western Australia 6009 Australia; 2 School of Medical and Health Sciences, Edith Cowan University, Joondalup, Western Australia 6027 Australia

**Keywords:** Atrial septal defect, Cannabis, Cannabinoid, Δ9-tetrahydrocannabinol, Cannabigerol, Cannabidiol, Mechanisms, Congenital anomalies, Cardiac malformations

## Abstract

**Background:**

Cardiovascular anomalies are the largest group of congenital anomalies and the major cause of death in young children, with various data linking rising atrial septal defect incidence (ASDI) with prenatal cannabis exposure. Objectives / Hypotheses. Is cannabis associated with ASDI in USA? Is this relationship causal?

**Methods:**

Geospatiotemporal cohort study, 1991–2016. Census populations of adults, babies, congenital anomalies, income and ethnicity. Drug exposure data on cigarettes, alcohol abuse, past month cannabis use, analgesia abuse and cocaine taken from National Survey of Drug Use and Health (78.9% response rate). Cannabinoid concentrations from Drug Enforcement Agency. Inverse probability weighted (ipw) regressions. Analysis conducted in R.

**Results:**

ASDI rose nationally three-fold from 27.4 to 82.8 / 10,000 births 1991–2014 during a period when tobacco and alcohol abuse were falling but cannabis was rising. States including Nevada, Kentucky, Mississippi and Tennessee had steeply rising epidemics (Time: Status β-estimate = 10.72 (95%C.I. 8.39–13.05), *P* < 2.0 × 10 ^− 16^). ASDI was positively related to exposure to cannabis and most cannabinoids.

Drug exposure data was near-complete from 2006 thus restricting spatial modelling from 2006 to 2014, *N* = 282. In geospatial regression models cannabis: alcohol abuse term was significant (β-estimate = 19.44 (9.11, 29.77), *P* = 2.2 × 10 ^− 4^); no ethnic or income factors survived model reduction. Cannabis legalization was associated with a higher ASDI (Time: Status β-estimate = 0.03 (0.01, 0.05), *P* = 1.1 × 10 ^-3).^ Weighted panel regression interactive terms including cannabis significant (from β-estimate = 1418, (1080.6, 1755.4), *P* = 7.3 × 10 ^-15).^ Robust generalized linear models utilizing inverse probability weighting interactive terms including cannabis appear (from β-estimate = 78.88, (64.38, 93.38), *P* = 1.1 × 10 ^-8).^ Marginal structural models with machine-aided SuperLearning association of ASDI with high v. low cannabis exposure R.R. = 1.32 (1.28, 1.36). Model e-values mostly > 1.5.

**Conclusions:**

ASDI is associated with cannabis use, frequency, intensity and legalization in a spatiotemporally significant manner, robust to socioeconomicodemographic adjustment and fulfilled causal criteria, consistent with multiple biological mechanisms and similar reports from Hawaii, Colorado, Canada and Australia. Not only are these results of concern in themselves, but they further imply that our list of the congenital teratology of cannabis is as yet incomplete, and highlight in particular cardiovascular toxicology of prenatal cannabinoid and drug exposure.

**Supplementary Information:**

The online version contains supplementary material available at 10.1186/s12887-020-02431-z.

## Introduction

Atrial Septal Defect (secundum type) (ASD) is one of the commonest of the cardiovascular congenital anomalies which are themselves the commonest form of congenital defect. Congenital defects are the commonest cause of mortality in children under the age of 5 years [1]. The Centres for Disease Control Atlanta, Georgia, (CDC) publishes rates of congenital anomalies across the USA annually based on reports from state-based registries through the National Births Defects Prevention Network which CDC sponsor. Review of these data indicate that in recent years ASD appears to be increasing in some US States for reasons which were not apparent.

Previous reports from Hawaii and Colorado had linked ASD with cannabis exposure [2, 3]. Indeed the report on Colorado showed that ASD incidence (ASDI) followed a sigmoidal trajectory and closely tracked the decade of cannabis legalization there [3]. Canada Health recently issued a major report of that nation’s teratological experience which noted a rise in total cardiovascular defects in the northern territories of Canada [4, 5] which are known to consume more cannabis [6]. Since ASD is one of the most common cardiovascular defects it is likely that ASD was represented in this general increase. Government reports from Australia similarly link high ASDI with areas of high cannabis use [7]. Moreover 34 congenital defects including nine cardiovascular defects were recently noted to be more common in the highest quintile of cannabis using states in the USA than in the remainder of the country [8].

A previous joint position statement from the American Academy of Pediatric and the American Heart Association linked prenatal cannabis exposure with ventricular septal defect and Ebsteins anomaly [9]. The American College of Obstetricians and Gynaecologists and the Society of Obstetricians and Gynecologists of Canada recommend that women avoid the use of cannabis during pregnancy [10, 11]. However a detailed investigation of the association and its potentially causal relationship has not been reported.

The current study explored three nested hypotheses which were conceived before beginning the study. Firstly, was there indeed an increase in ASDI? Secondly, was the association robust to adjustment for definable sociodemographic, socioeconomic and drug exposure covariates across space and time? And thirdly, was the relationship causal? We were particularly interested to apply the powerful methods of formal geotemporospatial analysis and causal inference to these problems. If these hypotheses were confirmed this would raise the intriguing possibility that, notwithstanding statements from official authorities, our list of cannabis-associated birth anomalies remains incomplete, and there is more to learn in this important area.

## Methods

### Design

This study was a retrospective observational geotemporospatial epidemiological study of publicly available datasets looking at the relationships of ASD with drug exposure, ethnicity and socioeconomic data in USA 1989–2016. This study was performed in January 2020.

### Data

Data on birth defects was sourced from the National Birth Defect Prevention Network (NBDPN) annual reports 1988–1989 to 2012–2016 [12]. This report compiles the reports of the State Birth Defects registries in multi-year groups. The reference year for each report was the middle year of each report. Data on drug use was accessed from the National Survey of Drug Use and Health (NSDUH) conducted annually by the Substance Abuse and Mental Health Services Administration (SAMHSA) [13]. This is a nationally representative sample of the non-institutionalized US population. Data on five drugs was extracted at state level: last month: cigarette, alcohol abuse or dependence, and cannabis use; and last year: analgesic abuse and cocaine use. Data on cannabis use rates by ethnicity was also extracted at the national level. Data on cannabinoid concentration is the average concentration of the various cannabinoids found annually in Drug Enforcement Agency seizures [14, 15]. Data for the ethnic population of each state was sourced from the US Census Bureau’s decennial and 5 year annual community surveys via the tidycensus package in “R”.

### Derived variables

Frequency of cannabis use in the last month by ethnicity was sourced from the Substance Abuse and Mental Health Data Archive and used to calculate a mean number of days used at the Federal level for each year [16]. These measures were multiplied by the last month cannabis use for that state and then by the mean annual concentration of Δ9-tetrahydrocannabinol (THC) to derive an index of annual ethnic THC exposure at state level (AETES) referred to in the Tables as an ethnic “score”. This variable was used to standardize population ethnicity compositions for known different use rates and intensity of cannabis use.

### Statistics

This analysis was conducted in January 2020 using “R” Studio version 1.2.5042 based on “R” version 4.0.0 obtained from CRAN [17]. Variables were log-transformed guided by the Shapiro test. Data was matched and formatted using “R” package dplyr [18], maps and graphs were drawn in ggplot2 [18, 19] and sf [20], linear regression was performed in base, geofacetting was done in geofacet, panel regression was done in plm [21], geospatial linkages and weights were assigned in spdep [22], and Alaska and Hawaii were elided using albersusa and sp. Factor analysis was done with factoextra. Two-stage regression including instrumental variables (as indicated) was conducted in panel and geospatial regression. Model reduction was by the classical method of serial deletion of the least significant term. For non-spatial models missing data was casewise deleted. Missing data was imputed for spatial analysis by temporal kriging (mean substitution) as indicated. Spatial regression was performed in splm::spreml using a full model with Kapoor, Kelejian, and Prucha -type spatial errors, spatial lagging, random errors and serially autocorrelated errors (sem2srre + lag) in all cases [22–26]. Geospatial models were compared using the spatial Hausman test.

To balance confounding for measured covariates inverse probability weights over time (iptw) were computed for the kriged longitudinal data in a time-based paradigm (from package ipw [27]) and added to the dataset. Robust inferential analysis was conducted with iptw using mixed effects models (nlme), panel models (plm [21]) and generalized linear models (survey [28]). Marginal structural models were performed in doubly robust targeted minimum loss-based estimation (drtmle) which includes augmented-iptw. Adaptive machine learning was also conducted using drtmle to access SuperLearner libraries which heightened inferential power. Generalized linear models was used to specify model structure and machine learning was used to increase inferential sensitivity. eValues were computed (EValue [29–31]) to assess the required impact of unmeasured confounding. *P* < 0.05 was considered significant.

### Data availability statement

Data including software programming in R has been made publicly available through the Mendeley data repository at URL: 10.17632/vrnfbytrrr.1.

### Ethics

The study was approved by the Human Ethics Research Committee of the University of Western Australia April 1st 2019, No. RA/4/20/4724.

## Results

Across the NBDPN annual reports 1988–1989 to 2012–2016 (referred to as reference years 1989–2014) there were 347 reports of rates of atrial septal defect (secundum type). Across the period 2002–2016 the NSDUH was completed by 952,717 respondents out of 1,207,606 selected for the survey, a mean response rate of 78.89%. The survey quotes a mean weighted interview response rate of 74.1%.

The data for the 37 states contributing data are shown in eTable 1 and map-graphically in eFigure 1.

**Fig. 1:**
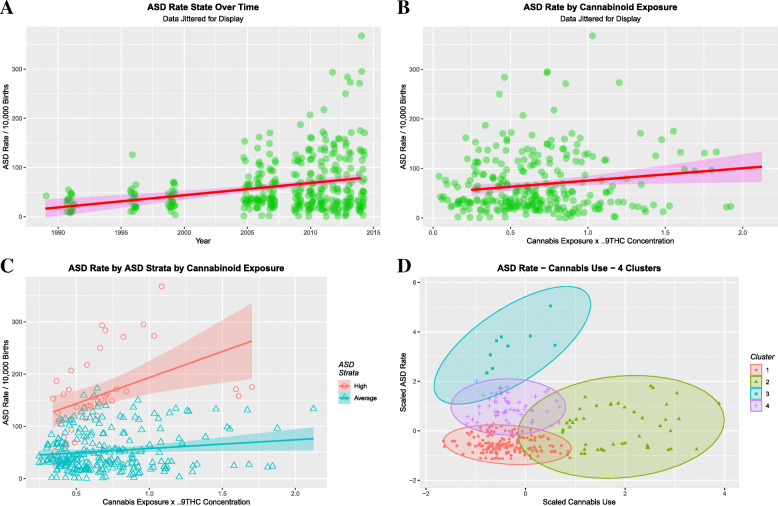
Univariate ASD trends. **a** ASDI over time. **b** ASDI by state THC exposure. **c** ASDI by THC exposure dichotomized into high and low ASDI states. **d** Cluster Analysis of the ASD – Cannabis exposure data

Figure 1a shows the time course of ASD across the USA and notes a rising trend. The mean rate in 1991 was 27.4/10,000 births and 82.4/10,000 in 2014, a 3.02-fold rise. Figure 1b plots the ASD Rate against the product of the last month cannabis use rate and the THC potency, and importantly, also shows an apparently rising trend with cannabis exposure. Close inspection of Fig. 1b shows that it seems to be bimodal with both upper and lower zones. When the highest ASD states from the 2012–2016 period, Nevada, Alaska, Mississippi, Tennessee, Ohio, Oregon and Kentucky are grouped together the appearance found in Fig. 1c is derived. eTable 2 quantitates these changes by linear regression and notes highly significant changes with time (β-estimate = 2.52, (95%C.I. 1.56, 3.48); with THC exposure index (β-estimate = 24.93, (1.06, 45.8)) and between the high and average ASD-rate states (Exposure: Status interaction (β-estimate = 10.72 (8.39–13.05), *P* < 2.2 × 10^− 16^). Figure 1d shows that formal cluster analysis correctly dissects out these zones. The reason for this bimodality is unclear, but may relate to local cannabinoid concentrations or intensity of use.

eFigure 2A shows the ASDI by cannabis consumption quintiles. An abrupt jump is noted between quintiles 3 and 4 shown as non-overlapping notches on the boxplots (ChiSq. for trend = 8147.9, df = 4, *P* < 10^− 300^). This is highlighted in eFig. 2B which dichotomizes the data around this point (Students-t = 3.48, df = 40.07, *P* = 0.0012). Ranges are provided in eTable 3 (Quintiles 1–3 45.46 (24.50, 93.30) (median, interquartile range) and Quintiles 4–5 94.70 (35.35, 175.20)).

eFigure 3 is a geofacetted plot of the ASDI by state across the USA with each state in approximately is actual position. Strongly rising trends are noted in Nevada, Kentucky, Tennessee, Mississippi, Missouri, Colorado and Alaska. eFigure 4 is a similar geofacetted plot of the ASDI this time against the THC exposure index. Curiously strongly rising trends are noted in states including Nevada, Kentucky, Mississippi and Tennessee.

**Fig. 2:**
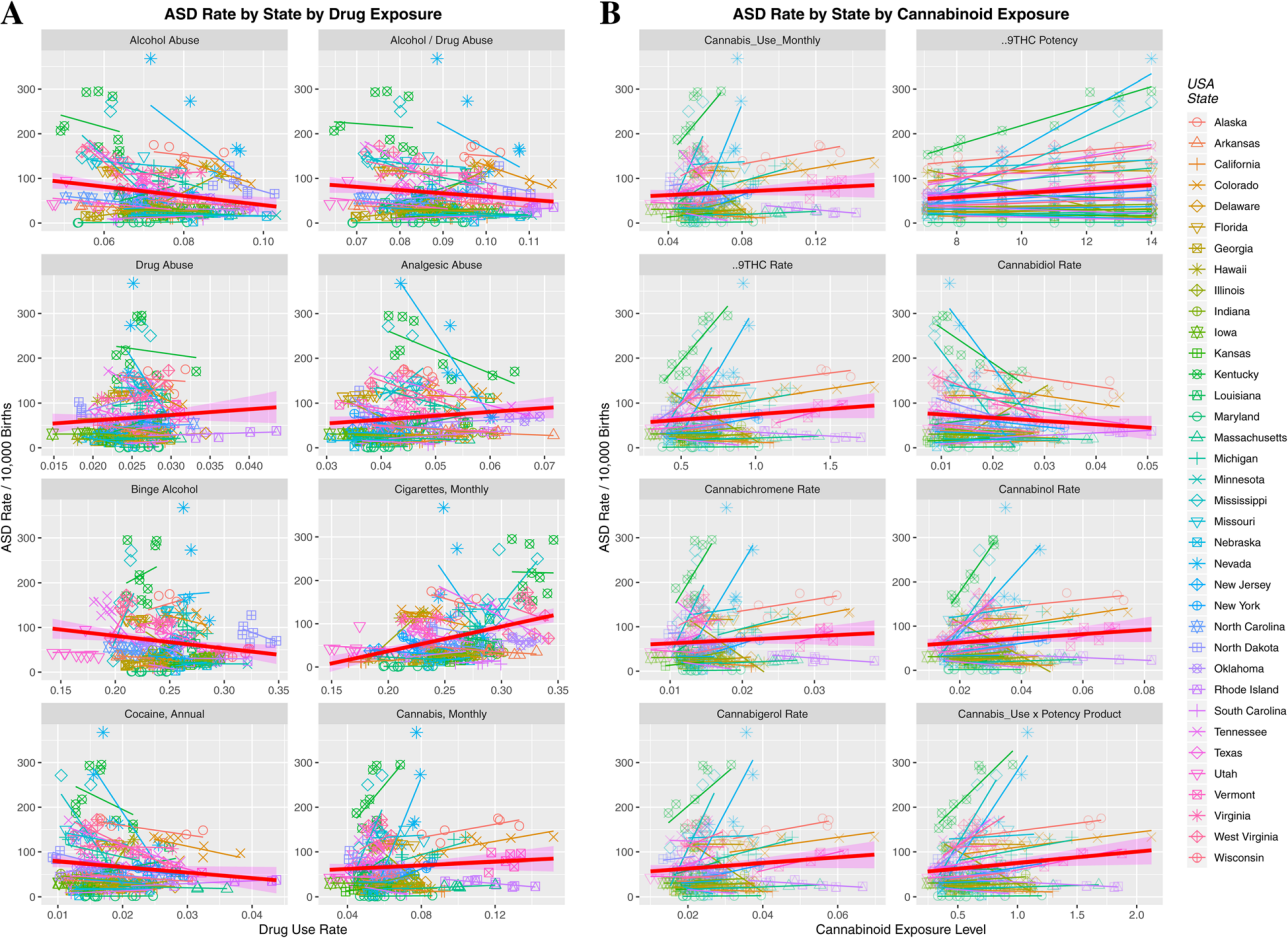
ASDI by (**a**) Drugs and (**b**) Cannabinoids by US State

Figure 2a shows the relationship of the ASDI to the drug exposure level in each state. Falling ASDI are noted with alcohol abuse, binge alcohol, cocaine and alcohol or drug abuse categories. Figure 2b is a similar illustration of the relationship of ASD to various cannabinoids but here one notes a rising relationship with most cannabinoids with the notable exception of cannabidiol.

eFigure 5A displays the rate of cannabinoid exposure by year and notes that for cannabidiol this has been a negative trend which makes interpretation of its relationship with ASD complex (Fig. 2b). Figure 5B is an annual plot of the ASD: cannabidiol relationship and notes a more positive relationship in 2006 and 2007 at a time when cannabidiol exposure levels were higher.

eFigure 6 charts the ASDI by the ethnic composition. Whilst the relationship is negative in the African-American community it appears to be positive amongst American Indians / Alaskan Natives (AIAN). As shown in eTable 4 these differences are significant.

When the ASDI is charted against the ethnic exposure to THC (AETES) as in eFigure 7, these ethnic differences disappear as uniform rising trends are seen.

As noted in eFigure 8 there is a non-significant relationship of ASDI with median household income.

These data may be regressed in their totality by panel regression which is a technique well suited to serial geospatial panel data with missing values. The results shown in eTable 5 are notable in that when median household income and racial composition are regressed by themselves they are not significant. When drugs, income and ethnicity are regressed together ethnicity and income remain in the final model but have negative β-coefficients, and terms including cannabis also remain in the final model (from β-estimate = 22.25 (10.13, 150.24), *P* = 3.8 × 10^− 4^).

**Fig. 3:**
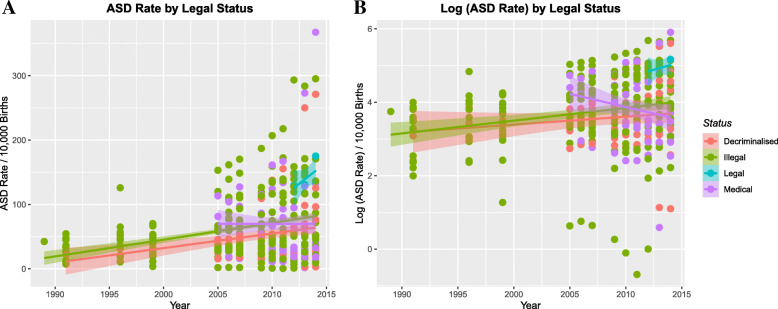
ASDI by legal status of cannabis. **a** Raw data plot. **b** log(ASDI) plot

When one charts the impact of the legal status of cannabis on the ASDI the data shown in Fig. 3 is obtained for the (A) raw and (B) log-transformed data respectively. As shown in eTable 6 the time:legal status interaction is highly significant (β-estimate = 0.03 (0.012, 0.48), *P* = 0.0011) for legal cannabis compared to other categories.

Missing data are not permitted in geospatial analytical techniques. Temporal kriging is an accepted method of completing such values. eTable 7 shows all the data with kriged values coloured. From 2006 when all the drug data is available there are 267 ASD datapoints. 29 (eTable 8) have been inserted by kriging bringing the total (eTable 9) to 296 points. The complete spatial dataset is map-graphed in eFigure 9.

The spatial links used to derive the spatial weights are shown in eFigure 10 (A) in edited and (B) in final format after conceptually eliding (moving) Alaska and Hawaii into relationship with California.

The outcomes of spatial regression are shown in Table 1. Median household income and demographics was not significant either with or without instrumental variables either alone or in combination (not shown). When the drugs alone were regressed the results shown in the upper part of the table were derived. When drugs and race and a full complement of instrumental variables was regressed the same model was returned with terms including cannabis continuing to be significant. When a drug-only model was regressed at 2 years lag the results shown were derived. When the procedure was repeated at 4 years of lag to account for the moving average style of data published by NBDPN the results were again as shown. In each model terms including cannabis remain significant in final models.

**Table 1:** Geotemporospatial Regression

Instrumental ± Lagged Variables	Parameters & Values			Model			
	Parameter	β-Coefficient (95%C.I.)	***P***-value	LogLik	Parameters	Value	P-Value
	***Drugs***						
	***0 Lags***						
	spreml(ASD_Rate ~ cigmon * mrjmon * abodalc + anlyr + cocyr)						
Δ9THC_Exposure	mrjmon	0.49 (0.16, 0.82)	0.0033	−56.071	phi	2.57E-05	1
Cannabigerol_Exposure	cocyr	−0.25 (− 0.43, − 0.07)	0.0085		psi	0.9629	<2e-16
	cigmon: mrjmon	−0.87 (−1.71, − 0.03)	0.0416		rho	− 0.0414	0.7944
					lambda	−0.0455	0.7702
	***Drugs & 4 Races***						
	***0 Lags***						
Δ9THC_Exposure	spreml(ASD_Rate ~ cigmon * mrjmon * abodalc + anlyr + cocyr + 4_Races)						
Cannabigerol_Exposure	mrjmon	0.49 (0.16, 0.82)	0.0033	−56.071	phi	2.57E-05	1
Caucasian_Daily_Score	cocyr	−0.25 (−0.43, − 0.07)	0.0085		psi	0.9629	<2e-16
African.Am_Daily_Score	cigmon: mrjmon	−0.87 (−1.71, − 0.03)	0.0416		rho	− 0.0414	0.7944
Asian.Am_Daily_Score					lambda	−0.0455	0.7702
AIAN_Daily_Score							
	***Drugs***						
	***2 Lags***						
	spreml(ASD_Rate ~ cigmon * mrjmon * abodalc + anlyr + cocyr)						
Δ9THC_Exposure, 0:2	mrjmon	0.46 (0.07, 0.85)	0.0237	− 73.445	phi	15.4679	7.26E-07
Cannabigerol_Exposure, 0:2					psi	0.6471	0.0002
					rho	−0.0352	0.8684
					lambda	−0.0667	0.7517
	***Drugs***						
	***4 Lags***						
	spreml(ASD_Rate ~ cigmon * mrjmon * abodalc + anlyr + cocyr)						
Δ9THC_Exposure, 0:4	mrjmon: abodalc	19.44 (9.11, 29.77)	0.0002	−55.133	phi	1.3947	NA
Cannabigerol_Exposure, 0:4	cigmon: mrjmon: abodalc	−45.14 (− 75.09, − 15.19)	0.0031		psi	0.9530	<2e-16
					rho	0.0204	0.9361
					lambda	−0.1420	0.5256

*Abbreviations*

*4_Races* Caucasian-American, African-American, Hispanic-American, Asian-American

*AIAN* American Indian / Alaska Native

Technical Notes:

*phi* Idiosyncratic component of the spatial error term

*psi* Individual time-invariant component of the spatial error term

*rho* Spatial autoregressive parameter

*lambda* Spatial autocorrelation coefficient

Models were compared. Based on their logLik values the first and fourth models are best. When these are directly compared spatial Hausman tests (ChiSq. = 11.18, df = 1, *P* = 8.25 × 10^− 4^) indicate the superiority of the drug model lagged to 4 years. One notes that in this model terms including cannabis were significant from (β-estimate = 19.44 (9.11, 29.77), *P* = 2.2 × 10^− 4^).

The kriged dataset may also be used to derive inverse probability weights (iptw). These are applied to panel models in eTable 10 with a marked gain in both power (F = 15.62 on df = 7280 to F = 1062.95, df = 13,274) and inferential sensitivity with interactive terms including cannabis significant (from β-estimate = 1418, (1080.6, 1755.4), *P* = 7.3 × 10^− 15^).

iptw can be applied to mixed models (eTable 11) where interactive terms including cannabis are significant (from β-estimate = 6.80, (5.86, 7.74), *P* < 10^− 4^).

iptw can be utilized in robust conditional generalized linear models (glm). Table 2 sets out these results in an additive model, and in interactive models with a four-way interaction in drug terms, a four-way interaction in ethnic cannabis exposure index, and an additive combination of two three way interactions between drugs and ethnic cannabis exposure. Highly significant interactive terms including cannabis appear (from β-estimate = 78.88, (64.38, 93.38), *P* = 1.1 × 10^− 8^).

**Table 2:** Robust General Linear Model with Inverse Probability Weights

Parameter	β-Coefficient (95%C.I.)	***P***-value
***Additive Model***		
AIAN.Amn	0.73 (0.46, 1)	2.8E-05
NHCauc.Amn_Cannabis	5.42 (2.42, 8.42)	0.0015
Analgesics	0.72 (0.21, 1.23)	0.0100
Asian_Cannabis	1 (0.24, 1.76)	0.0160
NHAfrc.Amn_Cannabis	−4.55 (−7.53, −1.57)	0.0060
Alcohol	−34.96 (−49.93, − 19.99)	0.0001
NHPI_Cannabis	− 0.3 (− 0.42, − 0.18)	9.0E-05
Cauc.Amn	− 2.89 (− 3.91, − 1.87)	8.9E-06
Hispanic_Cannabis	− 1.18 (− 1.59, − 0.77)	5.6E-06
Afrc.Amn	− 0.82 (− 1.09, − 0.55)	4.4E-06
***Interactive Models***		
Four-Way Interaction Amongst Drugs: Tobacco * Alcohol * Cannabis * Analgesics		
Cannabis	913.3 (597.54, 1229.06)	5.8E-05
Analgesics	842 (549.57, 1134.43)	6.1E-05
Cannabis: Analgesics	294.1 (188.73, 399.47)	8.3E-05
Cigarettes: Alcohol	114,400 (68,496.8, 160,303.2)	0.0002
NHCauc.Amn_Cannabis	5.68 (3.37, 7.99)	0.0003
Cigarettes: Alcohol: Cannabis	40,180 (23,672.88, 56,687.12)	0.0003
Cigarettes: Alcohol: Analgesics	36,720 (21,412.4, 52,027.6)	0.0003
Cigarettes: Alcohol: Cannabis: Analgesics	12,870 (7331.04, 18,408.96)	0.0005
Afrc.Amn	−0.47 (−0.88, −0.06)	0.0440
Cauc.Amn	−3.03 (−4.95, −1.11)	0.0080
NHPI_Cannabis	−0.28 (− 0.44, − 0.12)	0.0027
Hispanic_Cannabis	− 1.99 (− 3.01, − 0.97)	0.0020
Alcohol: Cannabis: Analgesics	− 3184 (− 4540.71, − 1827.29)	0.0004
Alcohol: Analgesics	− 9055 (− 12,759.4, − 5350.6)	0.0003
NHAfrc.Amn_Cannabis	−2.38 (− 3.36, − 1.4)	0.0003
Alcohol: Cannabis	− 9987 (− 14,012.84, − 5961.16)	0.0003
Alcohol	− 28,390 (− 39,456.16, − 17,323.84)	0.0002
Cigarettes: Cannabis: Analgesics	− 1191 (− 1619.85, − 762.15)	8.6E-05
Cigarettes: Analgesics	− 3418 (− 4618.11, − 2217.89)	6.8E-05
Cigarettes: Cannabis	− 3686 (− 4975.29, − 2396.71)	6.5E-05
Cigarettes	−10,550 (− 14,176, − 6924)	5.5E-05
Four-Way Interaction Amongst Ethnic_Cannabis: Cauc.Amn * Afrc.Amn * Asian.Amn * AIAN.Amn		
Asian_Cannabis	78.88 (64.38, 93.38)	1.1E-08
NHCauc.Amn_Cannabis: Asian_Cannabis	294.32 (235.62, 353.02)	3.5E-08
NHCauc.Amn_Cannabis	549.56 (436.25, 662.87)	5.5E-08
NHCauc.Amn_Cannabis: Asian_Cannabis: AIAN.Amn_Cannabis	51.74 (40.9, 62.58)	6.9E-08
NHAfrc.Amn_Cannabis: AIAN.Amn_Cannabis	51.34 (34.03, 68.65)	2.6E-05
Cigarettes	9.15 (5.41, 12.89)	0.0002
NHCauc.Amn_Cannabis: NHAfrc.Amn_Cannabis: Asian_Cannabis	6.72 (2.33, 11.11)	0.0084
Cocaine	−1.23 (−1.99, −0.47)	0.0060
Afrc.Amn	−0.59 (− 0.84, − 0.34)	0.0004
Alcohol	−31.75 (−45.31, − 18.19)	0.0003
Cauc.Amn	−2.69 (−3.65, − 1.73)	4.5E-05
AIAN.Amn_Cannabis	−122.94 (− 162.47, − 83.41)	1.6E-05
NHCauc.Amn_Cannabis: NHAfrc.Amn_Cannabis	−67.25 (−88.59, − 45.91)	1.3E-05
Asian_Cannabis: AIAN.Amn_Cannabis	−66.75 (− 85.02, − 48.48)	2.3E-06
NHAfrc.Amn_Cannabis: Asian_Cannabis: AIAN.Amn_Cannabis	−50.24 (−63.74, − 36.74)	1.8E-06
Hispanic_Cannabis	− 11.55 (− 14.35, − 8.75)	4.8E-07
NHAfrc.Amn_Cannabis: Asian_Cannabis	− 195.38 (− 233.99, − 156.77)	3.1E-08
NHAfrc.Amn_Cannabis	− 426.55 (− 505.56, − 347.54)	1.3E-08
NHCauc.Amn_Cannabis: NHAfrc.Amn_Cannabis: AIAN.Amn_Cannabis	−9.25 (− 10.96, −7.54)	1.2E-08
3 + 3 Way Interactions: Tobacco * Alcohol * Cannabis + Cauc.Amn * AIAN.Amn * Asian.Amn		
Cigarettes: Alcohol	3696.88 (2707.37, 4686.39)	1.5E-07
Analgesics	1.53 (1.1, 1.96)	4.3E-07
Cigarettes: Alcohol: Cannabis	1467.06 (997.23, 1936.89)	2.5E-06
Cannabis	28.63 (18.07, 39.19)	1.9E-05
AIAN.Amn	0.71 (0.44, 0.98)	5.0E-05
Cauc.Amn	−2.52 (−4.24, −0.8)	0.0083
Afrc.Amn	− 0.78 (−1.09, − 0.47)	4.0E-05
Alcohol: Cannabis	−368.51 (− 503.71, − 233.31)	1.8E-05
Cigarettes: Cannabis	−113.89 (− 151.25, −76.53)	3.6E-06
Cigarettes	− 288.27 (− 376.25, − 200.29)	1.2E-06
Alcohol	− 950.06 (− 1226.77, − 673.35)	5.8E-07

iptw can also be used in glm in drtmle models to formulate marginal structural models with both glm and adaptive machine learning algorithms using the above noted increment from the third to the fourth cannabis exposure quintile as a switch to signal high v. low dose cannabis exposure (Table 3).

**Table 3:** Doubly Robust Targeted Minimum Loss-Based Estimation Using Generalized Linear Models and SuperLearner Adaptive Machine Learning

Covariates	Model	Lower Exposure	Higher Exposure	R.R.	Difference w Higher Exposure	Wald Test, Z	***P***-Value
Cannabis_Monthly	Initial drtmle mrjmon SuperLearner Model	0.68 (0.61, 0.69)	0.76 (0.72, 0.80)	1.11 (1.05, 1.18)	0.08 (0.04, 0.13)	3.53	0.0004
Cigarettes	Binomial glm	0.68 (0.66, 0.69)	0.86 (0.84, 0.87)	1.26 (1.23, 1.29)	0.18 (0.16, 0.20)	17.16	5.02E-66
Alcohol_Abuse							
Cannabis_Monthly	SuperLearner	0.71 (0.69, 0.73)	0.98 (0.97, 0.98)	1.37 (1.34, 1.40)	0.27 (0.24, 0.29)	20.08	1.15E-89
Analgesic_Abuse							
Cocaine_Annual	Mixed SuperLearner - Glm Model Series						
% White							
% African-American	No Interactions						
% Asian	- Additive	0.68 (0.66, 0.70)	0.86 (0.84, 0.87)	1.26 (1.22, 1.30)	0.17 (0.16, 0.20)	17.16	5.03E-66
% American Indian / Alaskan Natives							
NHWhite_Daily_Cannabis_Use x THC_Concentration	One Interaction						
NHBlack_Daily_Cannabis_Use x THC_Concentration	- Tobacco x Cannabis	0.68 (0.66, 0.70)	0.82 (0.81, 0.84)	1.21 (1.18, 1.25)	0.14 (0.12, 0.16)	13.16	1.57E-39
NHAsian_Daily_Cannabis_Use x THC_Concentration							
NHAIAN_Daily_Cannabis_Use x THC_Concentration	Three Interactions						
NHHispanic_Daily_Cannabis_Use x THC_Concentration	- Tobacco * Cannabis * Alcohol	0.68 (0.66, 0.70)	0.78 (0.75, 0.82)	1.15 (0.10, 1.20)	0.10 (0.7, 0.14)	5.95	2.62E-09
Median_Household_Income							
	Four Substance Interactions						
	- Tobacco * Cannabis * Alcohol * Analgesics	0.68 (0.67, 0.70)	0.75 (0.71, 0.78)	1.08 (1.06, 1.11)	0.06 (0.02, 0.10)	2.85	0.0043
	Three Ethnic Interactions						
	NHWhite_Daily_Cannabis_Use x THC_Concentration *	0.68 (0.66, 0.70)	0.79 (0.75, 0.83)	1.16 (1.9, 1.24)	0.11 (0.06, 0.16)	4.72	7.74E-06
	NHAsian_Daily_Cannabis_Use x THC_Concentration *						
	NHAIAN_Daily_Cannabis_Use x THC_Concentration						
	Three Substance x Three Ethnic Interactions						
	- Tobacco * Cannabis * Alcohol *	0.70 (0.68, 0.72)	0.93 (0.92, 0.94)	1.32 (1.28, 1.36)	0.23 (0.21, 0.25)	18.52	1.32E-76
	NHWhite_Daily_Cannabis_Use x THC_Concentration *						
	NHAsian_Daily_Cannabis_Use x THC_Concentration *						
	NHAIAN_Daily_Cannabis_Use x THC_Concentration						

The top line shows that in a SuperLearner model cannabis exposure is significant alone with high v low exposure (0.08, (0.04, 0.13)).

Remaining models include all the covariates shown in the first column. Models become progressively more complex moving down the table. The most refined model is the final one which shows a marginal association of ASDI with high v. low cannabis exposure of 0.93 (0.92, 0.94) v. 0.70 (0.68, 0.72), a relative risk of 1.32 (1.28, 1.36), Wald Z = 18.52, *P* = 1.3 × 10^− 76^).

Sensitivity analyses may be conducted for these results using the eValue which quantitates the association an unobserved variable would have to have with both the measured parameter and the outcome to obviate the results. eTable 12 presents a variety of analyses with many results significantly divergent from unity making uncontrolled confounding unlikely across all major models and all major findings.

## Discussion

This is the first study to our knowledge to use geotemporospatial and formal inferential analysis to assess the association between cannabis use and ASDI. Data confirm a positive relationship between cannabis use and ASDI. Specifically, we firstly confirmed that ASDI increased three-fold 1989–2016. Notably this ASDI elevation is largely accounted for by states such as Nevada, Kentucky, Mississippi and Tennessee and associated with the legalization of cannabis. Second, increases in ASDI occurred over a period when alcohol, tobacco and cocaine use were falling, which implicates rising cannabis exposure [32]. Cannabis legalization was associated with higher ASDI.

In multivariable geotemporospatial regression the cannabinoid-ASDI link was confirmed across space and time together and was robust to adjustment for other socioeconomic and ethnodemographic variables.

Formal investigation of the cannabinoid-ASDI link by the tools of causal inference including by inverse probability time-based weighting in robust generalized linear models, doubly robust augmented inverse probability weighting and machine SuperLearning techniques confirmed the link and confirmed its robustness to adjustment for other measured variables in pseudorandomized populations. Sensitivity analysis utilizing evalues confirmed that unmeasured variables were unlikely to account for the size of the effect observed.

This combination of geotemporal analysis, the iptw inferential analysis and the sensitivity analysis in the context of the preliminary concordant trends together make a powerful combined epidemiological argument for a causal relationship between cannabinoid exposure and ASDI.

Hence this study made affirmative findings in relation to all three opening hypotheses. ASDI is indeed rising; drug and cannabinoid exposure account for this in a manner robust to adjustment for the usual socioeconomicodemographic covariates in final geospatial models; and finally the cannabinoid-ASDI relationship fulfills criteria for causality.

The association of cannabis use with atrial septal defect was first described in a report from Hawaii where its use in isolation was noted to be linked to an increase in ASDI of 6.12-fold (95%C.I. 1.98–14.35) [2]. It is interesting that many of these findings were also recently observed in Colorado [3]. The pronounced increase in ASDI across the decade of cannabis legalization there was noted above. Similar findings were also seen in relation to cannabidiol.

One important issue to consider is the existence of biological pathways from cannabis exposure to cardiovascular embryogenesis. Embryologically the heart forms from a complex series of sources including the primary and secondary heart fields, the proepicardium and migration of cells from the neural crest [33]. Many molecular cascades are involved including retinoic acid, the core regulatory network of MEF2, NKX2, GATA, Tbx, and Hand and various micro-RNA’s, and later TGFβ, BMP’s and notch become key molecular organizers. Genetic defects of Nkx 2–5, GATA4, Tbx5 and Downs syndrome are known to be linked with ASD pathogenesis [33].

Cannabinoid type 1 receptors (CB1R) are found in the embryo from the twelfth week of foetal life and exist in high density on the endocardial cushion material [34–37]. It has been noted that cannabinoids can act on the cardiovasculature via at least seven different receptors including the type 1 and 2 cannabinoid receptors, vanilloid receptors, GPR55, PPAR, abnormal cannabidiol receptors and others [38]. On occasion cannabinoids are known to induce proinflammatory states including arteritis and angiopathies [36, 37, 39–42]. Moreover in all seven studies to have examined the issue cannabis has been linked with gastroschisis which is believed to have a largely vasculopathic origin due to the implication of several vasoactive drugs, with a secondary defect in the right side of the abdominal wall arising due interference with the vascular supply. Hence there would appear to be a number of biological pathways potentially linking prenatal cannabis exposure with downstream adverse embryological cardiovascular outcomes.

This notable convergence of evidence linking cannabis exposure in USA, Colorado, Hawaii, Canada and Australia with biologically plausible pathways implies that our usual list of cannabis-related birth defects is as yet incomplete [2–4, 7]. Such an acknowledgement also raises the question of how many other birth defects are attributable to currently unidentified environmental causes.

The dramatic and recent rises in ASDI in some states is of particular concern. We feel that one possible explanation for the apparently bimodal response to THC exposure in the high ASD states may be an increase in the intensity of use or rising local cannabinoid potency, which is apparently not being well detected in NSDUH, which does not reveal publicly near daily cannabis consumption on a state basis. It is possible that changed agricultural arrangements in states previously majoring in tobacco cultivation with crop diversification into hemp products may explain some of the effects in Midwestern states. That this metric is apparently not being picked up in NSUDH is a matter for further investigation.

Study strengths include the use of US national census and American Community Survey, and nationally representative drug use surveys. One of the great advantages of conducting the present analysis on USA data is that it represents the most comprehensive data set globally. Secondly, this study represents the first use of formal geotemporospatial and causal inferential analytical techniques to assess the cannabis-ASD relationship. Thirdly, results are consistent with other reports and research from a range of jurisdictions which have used different methodologies and likely have had a range of different and likely potential confounders. Study limitations are firstly, data employed ecological aggregate-level data, which do not related to any specific individual; that is although we can say that State increased level of cannabis use correlated with same State increased rates of congenital anomalies we cannot specifically attribute a case of ASD to prenatal cannabis exposure from parents shown to be using high level cannabis. High intensity use of cannabis at state level is also not captured in the publicly available NSDUH data. Moreover congenital anomaly data from certain high cannabis use states including Washington, Oregon, Vermont and Maine are incomplete in many years which would weaken our estimates downwards. Secondly, utilised drug use data relied on self-report, the accuracy of which is difficult to validate. Thirdly, although we attempted to adjust for socioeconomic variables including tobacco, alcohol, and cocaine that may be routinely used by consumers of cannabis, it is not possible to completely remove confounding from observational data.

The replication of major findings in several geographically independent locations globally [2–4, 7, 9, 43, 44] suggest that the findings are widely generalizable. There are now well established biological mechanisms by which cannabis might increase the likelihood of ASD. Collectively therefore these diverse and converging sets of information support the results of the geotemporal and causal inferential analyses.

It is appears that the medical, political and broader community are yet to fully comprehend or appreciate the higher rate of birth defects associated with cannabis legalization. The present report indicates this remains an open question which warrants further vigorous research and careful investigation, for example at higher geospatial resolution and using patient-matched data and validated biomarker approaches [45] and at the molecular, cellular and epigenetic levels.

## Supplementary Information

**Additional file 1: eTable 1.** ASD Rate Data. **eTable 2.** ASD Rate – Linear Regressions. **eTable 3.** ASD Rate by Cannabis Use Quintile Groupings. **eTable 4.** Impact of Ethnicity over time on ASD Rate. **eTable 5.** Panel Regression Raw Dataset. **eTable 6.** Impact of Cannabis Legal Paradigm on ASD Rate. **eTable 7.** Kriged Data Input. **eTable 8.** Inserted Data for Kriging. **eTable 9.** Kriged Data Input for Geospatial and IPW Analysis. **eTable 10.** Unweighted and Weighted Panel Regressions on Kriged Data. **eTable 11.** Mixed Effects Model with Inverse Probability Weighting. **eTable 12.** eValue Sensitivity Analysis.

**Additional file 2: eFigure 1.** Map-graph of log (ASDI) by state over time. This graph is original.

**Additional file 3: eFigure 2.** ASDI by (A) quintiles and (B) by quintiles dichotomized as quintiles 1, 2 and 3 v. quintiles 4 and 5.

**Additional file 4: eFigure 3.** Geofacetted plot with each US state in approximately their appropriate position, showing the ASDI over time for each state.

**Additional file 5: eFigure 4.** Geofacetted plot with each US state in approximately their appropriate position, showing the ASDI over a denominator of the product of state level cannabis use by national THC concentration (which thus provides an estimate of the state level THC exposure).

**Additional file 6: eFigure 5.** Concentration of cannabinoids by state across time. National averaged trend line is shown in each case. (A) Selected cannabinoids with loess (localized polynomial) fitted curves. (B) Linear regression lines for state level cannabidiol exposure.

**Additional file 7: eFigure 6.** ASD rate by ethnicity by state.

**Additional file 8: eFigure 7.** ASD rate by ethnicity collated for the whole nation (A) by individual ethnicity and (B) for all ethnicities pooled.

**Additional file 9: eFigure 8.** ASD rate as a function of the median household income.

**Additional file 10: eFigure 9.** Map-graphs depicting the log (ASDI) by state by year for the temporally kriged dataset. These maps are originals.

**Additional file 11: eFigure 10.** Geospatial links (A) edited and (B) final used in geospatiotemporal modelling. These maps are originals.

## Data Availability

All data generated or analysed during this study are included in this published article and its supplementary information files. Data has been made publicly available on the Mendeley Database Repository and can be accessed from this URL 10.17632/p7myt3fbzs.1

## Abbreviations

AIAN: American Indian / Alaska Native
AETES: Annual Ethnic THC Exposure at State Level
Amn: American
ASD: Atrial septal defect (secundum type)
ASDI: Atrial septal defect incidence
BMP: Bone morphogenetic protein
CRAN: Comprehensive “R” Archive Network
GATA: GATA Binding Protein 1
GPR55: G-Protein Receptor 55
Hand: Heart and Neural Crest Derivatives Expressed Proteins
lambda: Spatial autocorrelation coefficient
MEF2: Myocyte Enhancer Factor 2
NBDPN: National Birth Defects Prevention Network
NH: Non-Hispanic
NHPI: Non-Hispanic Pacific Islander
NKX2: NK2 Homeobox-1
NSDUH: National Survey of Drug Use and Health
phi: Idiosyncratic component of the spatial error term
PPAR: Peroxisome Proliferator-Activator Receptor
psi: Individual time-invariant component of the spatial error term
4_Races: Caucasian-American, African-American, Hispanic-American, Asian-American
rho: Spatial autoregressive parameter
SAMHSA: Substance Abuse and Mental Health Services Administration
Tbx: T-Box transcription factor
TGFβ: Transforming Growth Factor-β
THC: Δ9-Tetrahydrocannabinol
