# Supplementary material for: Contemporary epidemiology of rising atrial septal defect trends across USA 1991–2016: a combined ecological geospatiotemporal and causal inferential study
Source: BMC Pediatr. 2020 Nov 30;20:539. doi: 10.1186/s12887-020-02431-z (PMC7702707; doi:10.1186/s12887-020-02431-z)
Supplement: Supplementary file 1 — Additional file 1: eTable 1. ASD Rate Data. eTable 2. ASD Rate – Linear Regressions. eTable 3. ASD Rate by Cannabis Use Quintile Groupings. eTable 4. Impact of Ethnicity over time on ASD Rate. eTable 5. Panel Regression Raw Dataset. eTable 6. Impact of Cannabis Legal Paradigm on ASD Rate. eTable 7. Kriged Data Input. eTable 8. Inserted Data for Kriging. eTable 9. Kriged Data Input for Geospatial and IPW Analysis. eTable 10. Unweighted and Weighted Panel Regressions on Kriged Data. eTable 11. Mixed Effects Model with Inverse Probability Weighting. eTable 12. eValue Sensitivity Analysis. [file 12887_2020_2431_MOESM1_ESM.docx]

**eTables**

**eTable 1.: ASD Rate Data**

| State | 1989 | 1991 | 1996 | 1999 | 2005 | 2006 | 2007 | 2009 | 2010 | 2011 | 2012 | 2013 | 2014 |
| --- | --- | --- | --- | --- | --- | --- | --- | --- | --- | --- | --- | --- | --- |
| Colorado |  | 32.6 | 47.02 | 46.27 | 81.21 | 87.54 | 97.2 | 112.8 | 124.1 | 134 | 129.8 | 130.9 | 133.2 |
| Georgia |  | 44.92 | 56.72 | 25.32 | 37 | 32.44 | 27.5 | 14.5 | 13.9 | 14.7 | 16.1 | 17.8 | 18.7 |
| Illinois |  | 9.16 | 18.71 | 19.71 | 29.28 | 26.96 | 24.5 | 23.7 | 24.7 | 26.7 | 27.8 | 30.1 | 32.3 |
| Iowa |  | 26.77 | 20.89 | 21.9 | 31.03 | 31.71 | 30.5 | 30.9 | 30.3 | 31.2 | 31.9 | 30.9 | 29.4 |
| Kentucky |  |  |  | 19.71 | 153.06 | 161.59 | 170.3 | 187 | 206.9 | 217.6 | 293.4 | 283.9 | 295.3 |
| Louisiana |  |  |  |  |  | 111.13 | 55.1 | 57.3 | 45.2 | 34.4 | 54.5 | 63.2 | 71.9 |
| Maryland |  |  |  |  | 1.88 | 2.13 | 1.9 | 1.3 | 0.9 | 0.5 | 1 | 1.8 | 3 |
| Massachusetts |  | 7.34 | 11.08 | 11.25 | 17.87 | 18.02 | 19 | 20.5 | 22.4 | 22.6 | 22.8 | 24.2 | 26.4 |
| Michigan |  |  |  | 51.45 |  | 87.88 | 93.8 | 86.4 | 87.2 | 93.1 | 95.1 | 128.1 | 132.8 |
| Mississippi |  |  |  |  | 25.05 | 37.66 | 52.3 | 109.2 | 137.6 | 155.5 | 145.8 | 250.1 | 271.2 |
| Nebraska |  | 34.01 | 47.83 |  | 15.45 | 17.2 | 22.2 | 26.6 | 22.7 | 16.7 | 11.7 | 3.1 | 13 |
| New Jersey |  | 25.07 | 64.9 | 70.29 | 33.57 | 33.26 | 32 | 30.7 | 29 | 29.7 | 33.6 | 37.3 | 40.6 |
| New York |  | 21.89 | 36.15 | 29.08 | 45.46 | 45.99 | 45.8 | 47.9 | 52.2 | 55.5 | 64.3 | 69.6 | 76.5 |
| North Carolina |  | 11.14 | 31.13 | 33.01 | 37.7 | 37.6 | 40.6 | 51.7 | 54 | 56.5 | 56.3 | 54.3 | 51.1 |
| Oklahoma |  | 47.9 | 52.32 | 46.4 | 67.02 | 59.38 | 68 | 69.8 | 66.7 | 62.2 | 56.5 | 49.5 | 45.9 |
| Puerto Rico |  |  |  |  | 18.9 | 21.35 | 22.6 | 23.1 | 24 | 24.8 | 26.9 | 28.5 | 27.5 |
| Rhode Island |  | 54.43 |  |  | 34.62 | 35.35 | 37.9 | 37.2 | 32.1 | 27.3 | 24.4 | 23.5 | 22 |
| Tennessee |  | 12.64 | 10.64 |  | 62.51 | 78.69 | 93.3 | 126.4 | 136.4 | 139.5 | 148.6 | 157.2 | 170.7 |
| Texas |  |  |  | 39.56 | 58.27 | 62.53 | 64.7 | 65.6 | 74.9 | 79.3 | 82.8 | 85.5 | 86.7 |
| Utah |  |  |  | 3.56 | 33.7 | 35.94 | 37.7 | 43.1 | 42.9 | 42.7 | 94.7 | 39.2 | 35.2 |
| West Virginia |  |  |  |  | 66.33 | 76.35 | 75.8 | 119.1 | 159.4 | 172.4 | 158.7 | 144.1 | 136.4 |
| Wisconsin |  | 10.57 | 19.68 | 19.36 |  | 33.89 | 37 | 42.6 | 46.4 | 48.2 | 50.6 | 52.4 | 52.4 |
| California |  |  |  |  |  | 19.09 | 15.9 | 13.7 | 11.1 | 11.1 | 12.9 |  | 12.6 |
| Delaware |  |  |  | 41.43 |  |  | 22.3 | 24.2 | 24.2 | 27 | 29.7 | 30.5 | 32.3 |
| Minnesota |  |  |  |  |  |  | 17.7 | 16.8 | 16 | 17.2 | 19.9 | 22.5 | 21.1 |
| Nevada |  |  |  |  |  | 67.82 | 68.9 | 115.2 | 161.3 | 167.1 |  | 273.1 | 367.6 |
| Virginia |  | 11 | 18.8 | 25.12 |  | 107.06 | 110.3 | 113.4 | 116.9 | 111.9 | 105.2 | 116.5 |  |
| Arkansas | 42.37 | 36.29 | 36.15 | 30.83 | 27.69 | 28 | 35.2 | 34.7 | 38.8 | 37.4 |  | 36.6 |  |
| Kansas |  |  |  |  |  |  |  | 22.4 | 17.6 | 15.2 | 11.7 | 16 | 21.5 |
| Missouri |  | 33.67 | 21.29 | 66.53 |  |  | 114.6 |  | 134.2 | 136.9 | 139.4 | 149.9 | 125.4 |
| North Dakota |  |  |  |  | 65.31 | 65.71 | 70.2 | 84.1 | 88.1 | 102.4 | 127.5 |  |  |
| South Carolina |  |  |  |  |  |  | 23.8 | 9 | 15.6 |  | 6.9 | 8.4 | 9.2 |
| Vermont |  |  |  |  |  |  |  | 54.4 | 67.6 | 79.6 | 94.7 | 98.4 | 96.4 |
| Alaska |  |  | 44.82 | 68.82 | 120.17 | 139.16 | 148.6 |  |  |  | 158.3 | 171.1 | 175.2 |
| Florida |  |  |  | 53.41 |  |  |  |  | 116 | 117.7 | 119.4 | 117.4 | 114.9 |
| Hawaii |  | 46.43 | 125.88 | 22.79 | 113.31 | 104.08 | 127.2 |  |  |  | 18.5 | 18.4 | 18.4 |
| Indiana |  |  |  |  | 34.33 | 30.12 | 34.1 |  | 52 |  |  | 26.1 | 49.9 |

**eTable 2: ASD Rate – Linear Regressions**

| **Parameter** | **β-Coefficient (95%C.I.)** | **P-value** |
| --- | --- | --- |
|  |  |  |
| ***lm(ASD_Rate ~ Year)*** |  |  |
| Year | 2.52 (1.56, 3.48) | 4.2E-07 |
|  |  |  |
| ***lm(ASD_Rate ~ CanPot)*** |  |  |
| Cannabis_x_THC_Potency | 24.93 (4.06, 45.8) | 0.0199 |
|  |  |  |
| ***lm(ASD_Rate ~* Cannabis_x_THC_Potency *** ASD_High*)*** | | |
| Cannabis_x_THC_Potency: ASD_High_States | 83.1 (38.59, 127.61) | 0.0003 |
| ASD_High_States | 52.58 (19.26, 85.9) | 0.0022 |
| Cannabis_x_THC_Potency | 16.59 (-0.36, 33.54) | 0.0561 |
|  |  |  |
| ***lm(ASD_Rate ~ Year * fASD)*** |  |  |
| Year | 1.24 (0.51, 1.97) | 0.0009 |
| ASD_High_States | -21420 (-26083, -16757) | <2.0E-16 |
| Year: ASD_High_States | 10.72 (8.39, 13.05) | <2.0E-16 |

Abbreviations:

Cannabis - Last Month Cannabis Use

Cannabis_x_THC_Potency - Product of Last Month Cannabis Use

And THC Potency

ASD_High_State - ASD High States of :

Nevada,

Alaska,

Mississippi,

Tennessee,

Ohio,

Oregon

Kentucky

**eTable 3.: ASD Rate by**

**Cannabis Use Quintile Groupings**

| **Group** | **Limits** | **Sample Size** | **Median (IQR)** |
| --- | --- | --- | --- |
|  |  |  |  |
| Quintile 1 | 3.04 - 5.46% | 96 | 42.80 (29.34, 84.80) |
| Quintile 2 | 5.46 - 7.89% | 129 | 46.40 (26.60, 107.06) |
| Quintile 3 | 7.89 - 10.30% | 32 | 33.46 (18.40, 94.10) |
| Quintile 4 | 10.30 - 12.70% | 16 | 89.00 (36.28, 131.30) |
| Quintile 5 | 12.70 - 15.20% | 9 | 94.70 (24.40, 158.3) |
|  |  |  |  |
| Quintiles 1-3 | 3.04 - 10.30% | 257 | 45.46 (24.50, 93.30) |
| Quintiles 4-5 | 10.30 - 15.20% | 25 | 94.70 (35.35, 175.20) |

**eTable 4.: Impact of Ethnicity over time on ASD Rate**

| **Parameter** | **β-Coefficient (95%C.I.)** | **P-value** |
| --- | --- | --- |
|  |  |  |
| ***lm(ASD_Rate ~ CanPot)*** |  |  |
| Racial_THC_Exposure | 0.51 (0.12, 0.9) | 0.0103 |

**eTable 5.: Panel Regression Raw Dataset**

| **Instrumental + Lagged Variables** | **Parameter** | **β-Coefficient (95%C.I.)** | **P-value** |  |  |
| --- | --- | --- | --- | --- | --- |
|  |  |  |  |  |  |
|  | ***Drugs*** |  |  |  |  |
|  | ***0 Lags*** |  |  |  |  |
|  | ***plm(ASD_Rate ~ Cigarettes * Cannabis * Alcohol + Analgesics + Cocaine)*** | | | |  |
| THC_Exposure | Cigarettes | 94.8573 (44.77, 144.95) | 0.0002 | *** |  |
| CBG_Exposure | Cigarettes: Cannabis | 19.869 (7.22, 32.52) | 0.0021 | ** |  |
|  | Cannabis | -4.1948 (-7.28, -1.11) | 0.0078 | ** |  |
|  | Cigarettes: Alcohol | -405.8522 (-716.06, -95.65) | 0.0103 | * |  |
|  | Alcohol | 96.2582 (15.18, 177.34) | 0.0200 | * |  |
|  |  |  |  |  |  |
|  | ***2 Lags*** |  |  |  |  |
|  | ***plm(ASD_Rate ~ Cigarettes * Cannabis * Alcohol + Analgesics + Cocaine)*** | | | |  |
| mrjmon, 0:2 | Cigarettes | 21.01524 (13.15, 28.88) | 1.6E-07 | *** |  |
| THC_Exposure, 0:2 | Cigarettes: Cannabis | 3.02131 (0.87, 5.17) | 0.0059 | ** |  |
| CBG Exposure, 0:2 |  |  |  |  |  |
|  |  |  |  |  |  |
|  | ***4 Lags*** |  |  |  |  |
|  | ***plm(ASD_Rate ~ Cigarettes * Cannabis * Alcohol + Analgesics + Cocaine)*** | | | |  |
| mrjmon, 0:4 | Cigarettes: Alcohol | 15967.685 (2771.3, 29164.1) | 0.0177 | * |  |
| THC_Exposure, 0:4 | Cigarettes: Cannabis: Alcohol | 5715.927 (969.54, 10462.31) | 0.0183 | * |  |
| CBG Exposure, 0:4 | Cigarettes: Cannabis | -375.37 (-702.7, -48.04) | 0.0246 | * |  |
|  | Cigarettes | -1038.287 (-1953.9, -122.7) | 0.0262 | * |  |
|  | Alcohol | -3368.491 (-6348.4, -388.6) | 0.0267 | * |  |
|  | Cannabis: Alcohol | -1191.952 (-2266.6, -117.3) | 0.0297 | * |  |
|  | Cannabis | 78.769 (4.39, 153.15) | 0.0379 | * |  |
|  |  |  |  |  |  |
|  | ***Income*** |  |  |  |  |
|  | ***plm(ASD_Rate ~ Income)*** | | |  |  |
|  | Median Household Income | -0.0000171 (0, 0) | 0.002382 | ** |  |
|  |  |  |  |  |  |
|  | ***Races*** |  |  |  |  |
|  | ***0 Lags, Single Step Regression*** |  |  |  |  |
|  | ***plm(ASD_Rate ~Caucasian + African.Am. + AIAN + Asian.Pacific.Is)*** | | | | |
|  | AIAN | 0.194828 (0.01, 0.38) | 0.0359 | * |  |
|  | AsPI | 0.352518 (0.01, 0.69) | 0.0421 | * |  |
|  | White | 2.655111 (0.06, 5.25) | 0.0460 | * |  |
|  |  |  |  |  |  |
|  | ***Races*** |  |  |  |  |
|  | ***0 Lags, Two-Step Regression*** |  |  |  |  |
|  | ***plm(ASD_Rate ~Caucasian + African.Am. + AIAN + Asian.Pacific.Is)*** | | | | |
| Caucasian_Daily_Score | AIAN | 2.29316 (-75.37, 79.95) | 0.9538 |  |  |
| African.Am_Daily_Score | African.American | -2.23516 (-94.24, 89.77) | 0.9620 |  |  |
| Hispanic.Am_Daily_Score | Caucasian.American | -19.21097 (-1092.6, 1054.2) | 0.9720 |  |  |
| Asian.Am_Daily_Score | Asian.Pacific.Is.American | -0.59969 (-98.6, 97.4) | 0.9904 |  |  |
| AIAN_Daily_Score |  |  |  |  |  |
| NHPI_Daily_Score |  |  |  |  |  |
|  |  |  |  |  |  |
|  | ***Multi-Domain, 0 Lags*** |  |  |  |  |
|  | ***plm(ASD_Rate ~ Cigarettes * Cannabis * Alcohol + Analgesics + Cocaine +*** | | |  |  |
|  | ***MHY + Caucasian + African.Am. + AIAN + Asian.Pacific.Is)*** | | | |  |
|  | African.American | -0.451 (-0.64, -0.26) | 5.3E-06 | *** |  |
|  | Cigarettes | 102.7 (55.16, 150.24) | 3.1E-05 | *** |  |
|  | Cigarettes: Cannabis | 22.249 (10.13, 34.37) | 0.0004 | *** |  |
|  | MHY | -0.000031 (0, 0) | 0.0015 | ** |  |
|  | Cigarettes: Alcohol | -473.67 (-768.1, -179.3) | 0.0018 | ** |  |
|  | Cannabis | -4.2542 (-7.21, -1.3) | 0.0051 | ** |  |
|  | Alcohol | 110.34 (33.33, 187.35) | 0.0053 | ** |  |

**eTable 6.: Impact of Cannabis Legal Paradigm on ASD Rate**

| **Parameter** | **β-Coefficient (95%C.I.)** | **P-value** |
| --- | --- | --- |
|  |  |  |
| ***lm(ASD_Rate ~ Legal_Status*** |  |  |
| StatusLegal | 80.161 (25.11, 135.22) | 0.0046 |
|  |  |  |
| ***lm(ASD_Rate ~ Year * Legal_Status*** |  |  |
| Year:Legal_Status | 0.0301 (0.0123, 0.0479) | 0.0011 |
| Year:Medical_Status | 0.0296 (0.0118, 0.0474) | 0.0013 |
| Year:Illegal_Status | 0.0296 (0.0118, 0.0474) | 0.0013 |
| Year:Decriminalised_Status | 0.0295 (0.0117, 0.0473) | 0.0014 |

**eTable 7.: Kriged Data Input**

| State | 1989 | 1991 | 1996 | 1999 | 2005 | 2006 | 2007 | 2009 | 2010 | 2011 | 2012 | 2013 | 2014 |
| --- | --- | --- | --- | --- | --- | --- | --- | --- | --- | --- | --- | --- | --- |
| Colorado |  | 32.6 | 47.02 | 46.27 | 81.21 | 87.54 | 97.2 | 112.8 | 124.1 | 134 | 129.8 | 130.9 | 133.2 |
| Georgia |  | 44.92 | 56.72 | 25.32 | 37 | 32.44 | 27.5 | 14.5 | 13.9 | 14.7 | 16.1 | 17.8 | 18.7 |
| Illinois |  | 9.16 | 18.71 | 19.71 | 29.28 | 26.96 | 24.5 | 23.7 | 24.7 | 26.7 | 27.8 | 30.1 | 32.3 |
| Iowa |  | 26.77 | 20.89 | 21.9 | 31.03 | 31.71 | 30.5 | 30.9 | 30.3 | 31.2 | 31.9 | 30.9 | 29.4 |
| Kentucky |  |  |  | 19.71 | 153.06 | 161.59 | 170.3 | 187 | 206.9 | 217.6 | 293.4 | 283.9 | 295.3 |
| Louisiana |  |  |  |  |  | 111.13 | 55.1 | 57.3 | 45.2 | 34.4 | 54.5 | 63.2 | 71.9 |
| Maryland |  |  |  |  | 1.88 | 2.13 | 1.9 | 1.3 | 0.9 | 0.5 | 1 | 1.8 | 3 |
| Massachusetts |  | 7.34 | 11.08 | 11.25 | 17.87 | 18.02 | 19 | 20.5 | 22.4 | 22.6 | 22.8 | 24.2 | 26.4 |
| Michigan |  |  |  | 51.45 |  | 87.88 | 93.8 | 86.4 | 87.2 | 93.1 | 95.1 | 128.1 | 132.8 |
| Mississippi |  |  |  |  | 25.05 | 37.66 | 52.3 | 109.2 | 137.6 | 155.5 | 145.8 | 250.1 | 271.2 |
| Nebraska |  | 34.01 | 47.83 |  | 15.45 | 17.2 | 22.2 | 26.6 | 22.7 | 16.7 | 11.7 | 3.1 | 13 |
| New Jersey |  | 25.07 | 64.9 | 70.29 | 33.57 | 33.26 | 32 | 30.7 | 29 | 29.7 | 33.6 | 37.3 | 40.6 |
| New York |  | 21.89 | 36.15 | 29.08 | 45.46 | 45.99 | 45.8 | 47.9 | 52.2 | 55.5 | 64.3 | 69.6 | 76.5 |
| North Carolina |  | 11.14 | 31.13 | 33.01 | 37.7 | 37.6 | 40.6 | 51.7 | 54 | 56.5 | 56.3 | 54.3 | 51.1 |
| Oklahoma |  | 47.9 | 52.32 | 46.4 | 67.02 | 59.38 | 68 | 69.8 | 66.7 | 62.2 | 56.5 | 49.5 | 45.9 |
| Puerto Rico |  |  |  |  | 18.9 | 21.35 | 22.6 | 23.1 | 24 | 24.8 | 26.9 | 28.5 | 27.5 |
| Rhode Island |  | 54.43 |  |  | 34.62 | 35.35 | 37.9 | 37.2 | 32.1 | 27.3 | 24.4 | 23.5 | 22 |
| Tennessee |  | 12.64 | 10.64 |  | 62.51 | 78.69 | 93.3 | 126.4 | 136.4 | 139.5 | 148.6 | 157.2 | 170.7 |
| Texas |  |  |  | 39.56 | 58.27 | 62.53 | 64.7 | 65.6 | 74.9 | 79.3 | 82.8 | 85.5 | 86.7 |
| Utah |  |  |  | 3.56 | 33.7 | 35.94 | 37.7 | 43.1 | 42.9 | 42.7 | 94.7 | 39.2 | 35.2 |
| West Virginia |  |  |  |  | 66.33 | 76.35 | 75.8 | 119.1 | 159.4 | 172.4 | 158.7 | 144.1 | 136.4 |
| Wisconsin |  | 10.57 | 19.68 | 19.36 |  | 33.89 | 37 | 42.6 | 46.4 | 48.2 | 50.6 | 52.4 | 52.4 |
| California |  |  |  |  |  | 19.09 | 15.9 | 13.7 | 11.1 | 11.1 | 12.9 | 12.75 | 12.6 |
| Delaware |  |  |  | 41.43 |  | 28.0 | 22.3 | 24.2 | 24.2 | 27 | 29.7 | 30.5 | 32.3 |
| Minnesota |  |  |  |  |  | 17.7 | 17.7 | 16.8 | 16 | 17.2 | 19.9 | 22.5 | 21.1 |
| Nevada |  |  |  |  |  | 67.82 | 68.9 | 115.2 | 161.3 | 167.1 | 220.1 | 273.1 | 367.6 |
| Virginia |  | 11 | 18.8 | 25.12 |  | 107.06 | 110.3 | 113.4 | 116.9 | 111.9 | 105.2 | 116.5 | 116.5 |
| Arkansas | 42.37 | 36.29 | 36.15 | 30.83 | 27.69 | 28 | 35.2 | 34.7 | 38.8 | 37.4 | 37 | 36.6 | 36.6 |
| Kansas |  |  |  |  |  | 22.4 | 22.4 | 22.4 | 17.6 | 15.2 | 11.7 | 16 | 21.5 |
| Missouri |  | 33.67 | 21.29 | 66.53 |  | 114.6 | 114.6 | 124.4 | 134.2 | 136.9 | 139.4 | 149.9 | 125.4 |
| North Dakota |  |  |  |  | 65.31 | 65.71 | 70.2 | 84.1 | 88.1 | 102.4 | 127.5 | 127.5 | 127.5 |
| South Carolina |  |  |  |  |  | 23.8 | 23.8 | 9 | 15.6 | 11.25 | 6.9 | 8.4 | 9.2 |
| Vermont |  |  |  |  |  | 54.4 | 54.4 | 54.4 | 67.6 | 79.6 | 94.7 | 98.4 | 96.4 |
| Alaska |  |  | 44.82 | 68.82 | 120.17 | 139.16 | 148.6 | 151.0 | 158.3 | 165.5 | 158.3 | 171.1 | 175.2 |
| Florida |  |  |  | 53.41 |  | 116 | 116 | 116 | 116 | 117.7 | 119.4 | 117.4 | 114.9 |
| Hawaii |  | 46.43 | 125.88 | 22.79 | 113.31 | 104.08 | 127.2 | 100.0 | 72.8 | 45.6 | 18.5 | 18.4 | 18.4 |
| Indiana |  |  |  |  | 34.33 | 30.12 | 34.1 | 75.16 | 52 | 43.4 | 34.7 | 26.1 | 49.9 |

**eTable 8.: Inserted Data for Kriging**

| State | 2006 | 2007 | 2009 | 2010 | 2011 | 2012 | 2013 | 2014 | Count |
| --- | --- | --- | --- | --- | --- | --- | --- | --- | --- |
| California |  |  |  |  |  |  | 12.75 |  | 1 |
| Delaware | 28.0 |  |  |  |  |  |  |  | 1 |
| Minnesota | 17.7 |  |  |  |  |  |  |  | 1 |
| Nevada |  |  |  |  |  | 220.1 |  |  | 1 |
| Virginia |  |  |  |  |  |  |  | 116.5 | 1 |
| Arkansas |  |  |  |  |  | 37 |  | 36.6 | 2 |
| Kansas | 22.4 | 22.4 |  |  |  |  |  |  | 2 |
| Missouri | 114.6 |  | 124.4 |  |  |  |  |  | 2 |
| North Dakota |  |  |  |  |  |  | 127.5 | 127.5 | 2 |
| South Carolina | 23.8 |  |  |  | 11.25 |  |  |  | 2 |
| Vermont | 54.4 | 54.4 |  |  |  |  |  |  | 2 |
| Alaska |  |  | 151.0 | 158.3 | 165.5 |  |  |  | 3 |
| Florida | 116 | 116 | 116 |  |  |  |  |  | 3 |
| Hawaii |  |  | 100.0 | 72.8 | 45.6 |  |  |  | 3 |
| Indiana |  |  | 75.16 |  | 43.4 | 34.7 |  |  | 3 |
|  |  |  |  |  |  |  |  |  |  |
| Count Inserted | **7** | **3** | **5** | **2** | **4** | **3** | **2** | **3** | **29** |

**eTable 9.: Kriged Data Input for Geospatial and IPW Analysis**

| State | 2006 | 2007 | 2009 | 2010 | 2011 | 2012 | 2013 | 2014 |
| --- | --- | --- | --- | --- | --- | --- | --- | --- |
| Colorado | 87.54 | 97.2 | 112.8 | 124.1 | 134 | 129.8 | 130.9 | 133.2 |
| Georgia | 32.44 | 27.5 | 14.5 | 13.9 | 14.7 | 16.1 | 17.8 | 18.7 |
| Illinois | 26.96 | 24.5 | 23.7 | 24.7 | 26.7 | 27.8 | 30.1 | 32.3 |
| Iowa | 31.71 | 30.5 | 30.9 | 30.3 | 31.2 | 31.9 | 30.9 | 29.4 |
| Kentucky | 161.59 | 170.3 | 187 | 206.9 | 217.6 | 293.4 | 283.9 | 295.3 |
| Louisiana | 111.13 | 55.1 | 57.3 | 45.2 | 34.4 | 54.5 | 63.2 | 71.9 |
| Maryland | 2.13 | 1.9 | 1.3 | 0.9 | 0.5 | 1 | 1.8 | 3 |
| Massachusetts | 18.02 | 19 | 20.5 | 22.4 | 22.6 | 22.8 | 24.2 | 26.4 |
| Michigan | 87.88 | 93.8 | 86.4 | 87.2 | 93.1 | 95.1 | 128.1 | 132.8 |
| Mississippi | 37.66 | 52.3 | 109.2 | 137.6 | 155.5 | 145.8 | 250.1 | 271.2 |
| Nebraska | 17.2 | 22.2 | 26.6 | 22.7 | 16.7 | 11.7 | 3.1 | 13 |
| New Jersey | 33.26 | 32 | 30.7 | 29 | 29.7 | 33.6 | 37.3 | 40.6 |
| New York | 45.99 | 45.8 | 47.9 | 52.2 | 55.5 | 64.3 | 69.6 | 76.5 |
| North Carolina | 37.6 | 40.6 | 51.7 | 54 | 56.5 | 56.3 | 54.3 | 51.1 |
| Oklahoma | 59.38 | 68 | 69.8 | 66.7 | 62.2 | 56.5 | 49.5 | 45.9 |
| Puerto Rico | 21.35 | 22.6 | 23.1 | 24 | 24.8 | 26.9 | 28.5 | 27.5 |
| Rhode Island | 35.35 | 37.9 | 37.2 | 32.1 | 27.3 | 24.4 | 23.5 | 22 |
| Tennessee | 78.69 | 93.3 | 126.4 | 136.4 | 139.5 | 148.6 | 157.2 | 170.7 |
| Texas | 62.53 | 64.7 | 65.6 | 74.9 | 79.3 | 82.8 | 85.5 | 86.7 |
| Utah | 35.94 | 37.7 | 43.1 | 42.9 | 42.7 | 94.7 | 39.2 | 35.2 |
| West Virginia | 76.35 | 75.8 | 119.1 | 159.4 | 172.4 | 158.7 | 144.1 | 136.4 |
| Wisconsin | 33.89 | 37 | 42.6 | 46.4 | 48.2 | 50.6 | 52.4 | 52.4 |
| California | 19.09 | 15.9 | 13.7 | 11.1 | 11.1 | 12.9 | 12.75 | 12.6 |
| Delaware | 28.0 | 22.3 | 24.2 | 24.2 | 27 | 29.7 | 30.5 | 32.3 |
| Minnesota | 17.7 | 17.7 | 16.8 | 16 | 17.2 | 19.9 | 22.5 | 21.1 |
| Nevada | 67.82 | 68.9 | 115.2 | 161.3 | 167.1 | 220.1 | 273.1 | 367.6 |
| Virginia | 107.06 | 110.3 | 113.4 | 116.9 | 111.9 | 105.2 | 116.5 | 116.5 |
| Arkansas | 28 | 35.2 | 34.7 | 38.8 | 37.4 | 37 | 36.6 | 36.6 |
| Kansas | 22.4 | 22.4 | 22.4 | 17.6 | 15.2 | 11.7 | 16 | 21.5 |
| Missouri | 114.6 | 114.6 | 124.4 | 134.2 | 136.9 | 139.4 | 149.9 | 125.4 |
| North Dakota | 65.71 | 70.2 | 84.1 | 88.1 | 102.4 | 127.5 | 127.5 | 127.5 |
| South Carolina | 23.8 | 23.8 | 9 | 15.6 | 11.25 | 6.9 | 8.4 | 9.2 |
| Vermont | 54.4 | 54.4 | 54.4 | 67.6 | 79.6 | 94.7 | 98.4 | 96.4 |
| Alaska | 139.16 | 148.6 | 151.0 | 158.3 | 165.5 | 158.3 | 171.1 | 175.2 |
| Florida | 116 | 116 | 116 | 116 | 117.7 | 119.4 | 117.4 | 114.9 |
| Hawaii | 104.08 | 127.2 | 100.0 | 72.8 | 45.6 | 18.5 | 18.4 | 18.4 |
| Indiana | 30.12 | 34.1 | 75.16 | 52 | 43.4 | 34.7 | 26.1 | 49.9 |

**eTable 10.: Unweighted and Weighted Panel Regressions on Kriged Data**

| **Parameter** | **β-Coefficient (95%C.I.)** | **P-value** |
| --- | --- | --- |
|  |  |  |
| ***Unweighted Panel Model*** |  |  |
| Cigarettes | 98 (50.71, 145.29) | 6.3E-05 |
| Cigarettes: Cannabis | 22.01 (10.17, 33.85) | 3.2E-04 |
| Alcohol | 97.7 (19.18, 176.22) | 0.0154 |
| Cigarettes: Alcohol | -407.69 (-706.92, -108.46) | 0.0080 |
| Median_Household_Income | -1.45 (-2.47, -0.43) | 0.0055 |
| Cannabis | -4.29 (-7.21, -1.37) | 0.0043 |
| Afrc.Amn | -0.42 (-0.62, -0.22) | 3.5E-05 |
|  |  |  |
| ***Weighted Panel Model*** |  |  |
| Cigarettes: Alcohol | 4015.9 (3250.66, 4781.14) | < 2.2e-16 |
| AIAN.Amn | 0.6 (0.46, 0.74) | 2.6E-16 |
| Cigarettes: Cannabis: Alcohol | 1418 (1080.57, 1755.43) | 7.3E-15 |
| Cannabis | 29.6 (22.35, 36.85) | 3.8E-14 |
| Analgesics | 1.28 (0.91, 1.65) | 2.2E-10 |
| AsianPI.Amn | 0.45 (0.29, 0.61) | 4.2E-09 |
| Hispanic_Cannabis | 0.68 (0.27, 1.09) | 0.0013 |
| NHCauc.Amn_Cannabis | -0.91 (-1.46, -0.36) | 0.0013 |
| Cannabis: Alcohol | -344.96 (-438, -251.92) | 3.9E-12 |
| Cigarettes: Cannabis | -120.32 (-147.09, -93.55) | < 2.2e-16 |
| Alcohol | -1007.9 (-1213.56, -802.24) | < 2.2e-16 |
| Cigarettes | -340.62 (-406.79, -274.45) | < 2.2e-16 |
| Afrc.Amn | -0.61 (-0.73, -0.49) | < 2.2e-16 |

**eTable 11.: Mixed Effects Model with Inverse Probability Weighting**

| **Parameter** | **β-Coefficient (95%C.I.)** | **P-value** |
| --- | --- | --- |
|  |  |  |
| Cigarettes: Cannabis | 6.8 (5.86, 7.74) | <0.0001 |
| Analgesics | 1.21 (0.9, 1.52) | <0.0001 |
| MHY | 1.39 (1, 1.78) | <0.0001 |
| Cocaine | 0.88 (0.59, 1.17) | <0.0001 |
| NHCauc.Am_Cannabis | 2.22 (0.83, 3.61) | 0.0019 |
| White | 7.73 (0.18, 15.28) | 0.0460 |
| NHPacIsland.Amn_Cannabis | -0.11 (-0.21, -0.01) | 0.0246 |
| Cigarettes: Alcohol | -121.53 (-212.32, -30.74) | 0.0093 |
| NHAfrc.Am_Cannabis | -1.31 (-2.29, -0.33) | 0.0089 |
| AIAN | -2.59 (-3.81, -1.37) | <0.0001 |

**eTable 12.: eValue Sensitivity Analysis**

| **Parameter** | **Table** | **RR / Regression Coefficient (C.I.)** | **eValues** |
| --- | --- | --- | --- |
|  |  |  |  |
| ***Relative Risk*** |  |  |  |
| Quartiles 1-3 V 4-5 | eFigure 2 | 1.32 (1.28, 1.36) | 1.96, 1.87 |
|  |  |  |  |
| ***Linear Regression*** |  |  |  |
| ASD: Cannabis Relationship | eTable 2 | 24.93 (4.06, 45.8) | 2.66, 2.15 |
|  |  |  |  |
| Legal Relationship | eTable 6 | 80.16 (25.11, 135.22) | 2.82, 1.77 |
| Legal v Illegal Status | eTable 6 | 0.301 (0.012, 0.048) | 608.31, 384.96 |
|  |  |  |  |
| ***Mixed Effects iptw Regression*** |  |  |  |
| NHCauc.Amn_Cannabis_Use | eTable 11 | 2.22 (0.83, 3.61) | 2.89, 1.59 |
|  |  |  |  |
| ***Panel Regression - iptw*** |  |  |  |
| Hispanic_Cannabis Exposure | eTable 10 | 0.68 (0.27, 1.09) | 2.92, 1.52 |
|  |  |  |  |
| ***Geotemporospatial Regression*** |  |  |  |
| Cannabis : Alcohol | Table 1 | 19.44 (9.11, 29.77) | 3.02, 1.28 |
|  |  |  |  |
| ***Robust glm iptw Conditional Model*** |  |  |  |
| NHCauc.Amn_Cannabis_Use | Table 2 | 5.42 (2.42, 8.42) | 2.99, 1.36 |
|  |  |  |  |
| ***SuperLearner * GLM Interations*** |  |  |  |
| ***Marginal Structural Models*** |  |  |  |
| 3 Drugs + 3 Races Interactive | Table 3 | 1.32 (1.28, 1.36) | 1.96, 1.88 |
| Cannabis - ASD Assocation | Table 3 | 1.37 (1.34, 1.40) | 2.08, 2.014 |
