# Supplementary figures and images for: Contemporary epidemiology of rising atrial septal defect trends across USA 1991–2016: a combined ecological geospatiotemporal and causal inferential study
Source: BMC Pediatr. 2020 Nov 30;20:539. doi: 10.1186/s12887-020-02431-z (PMC7702707; doi:10.1186/s12887-020-02431-z)

**Log (ASD Rate) by State – Raw Data Plot**

Data: NBDPN Annual Reports

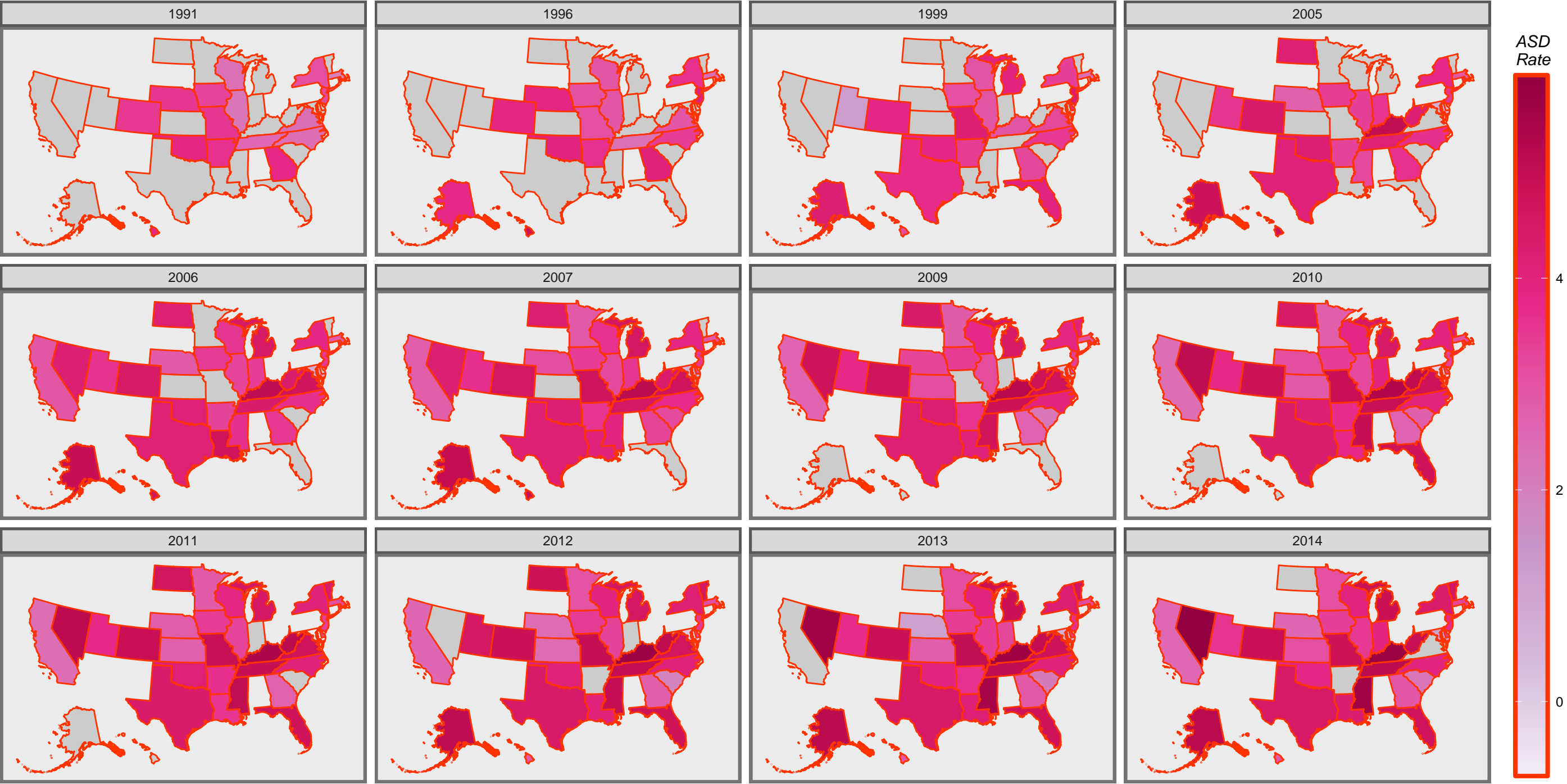

Supplement: Supplementary file 2 — Additional file 2: eFigure 1. Map-graph of log (ASDI) by state over time. This graph is original. [file 12887_2020_2431_MOESM2_ESM.pdf]

**Log (ASD Rate) by Quintiles  
– All Data**

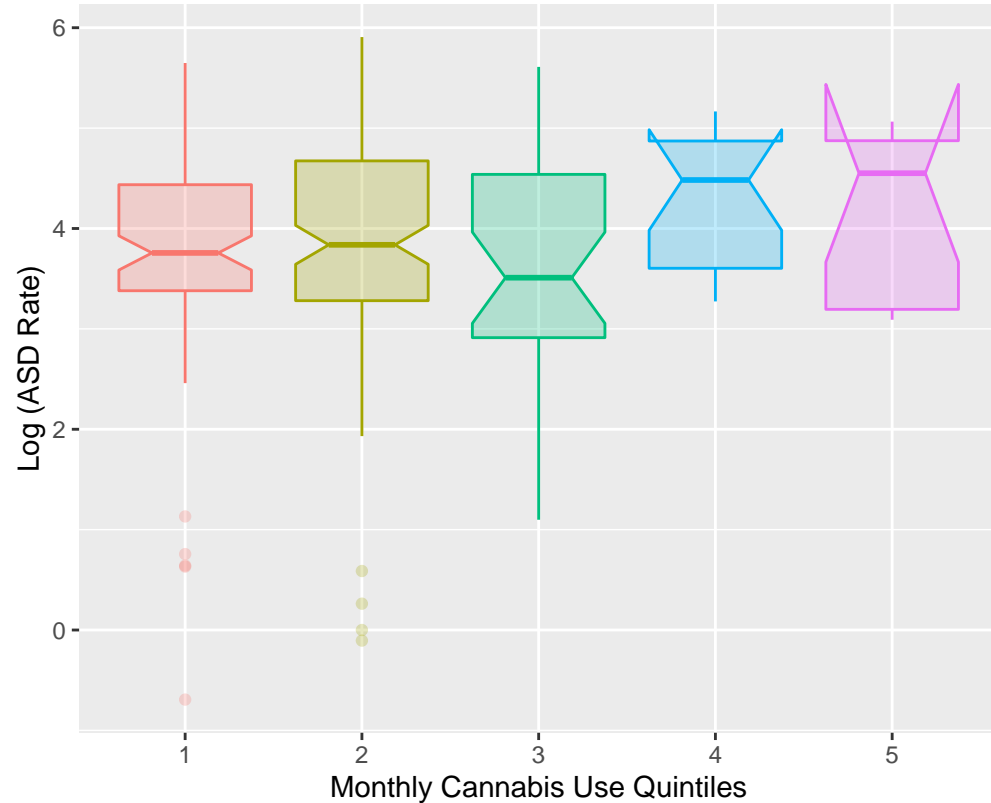

**Log (ASD Rate) by Quintiles 1, 2 & 3 Vs. Quintiles 4 & 5  
– All Data**

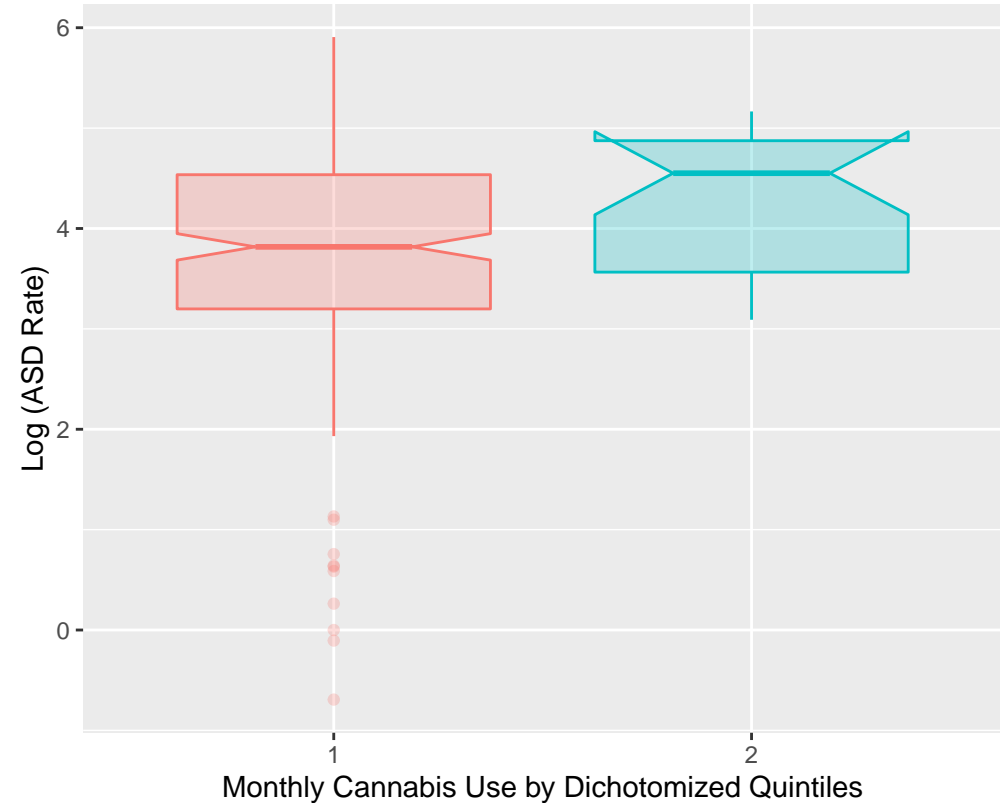

Supplement: Supplementary file 3 — Additional file 3: eFigure 2. ASDI by (A) quintiles and (B) by quintiles dichotomized as quintiles 1, 2 and 3 v. quintiles 4 and 5. [file 12887_2020_2431_MOESM3_ESM.pdf]

# ASD Rate by State by Cannabinoid Exposure – Regression Lines

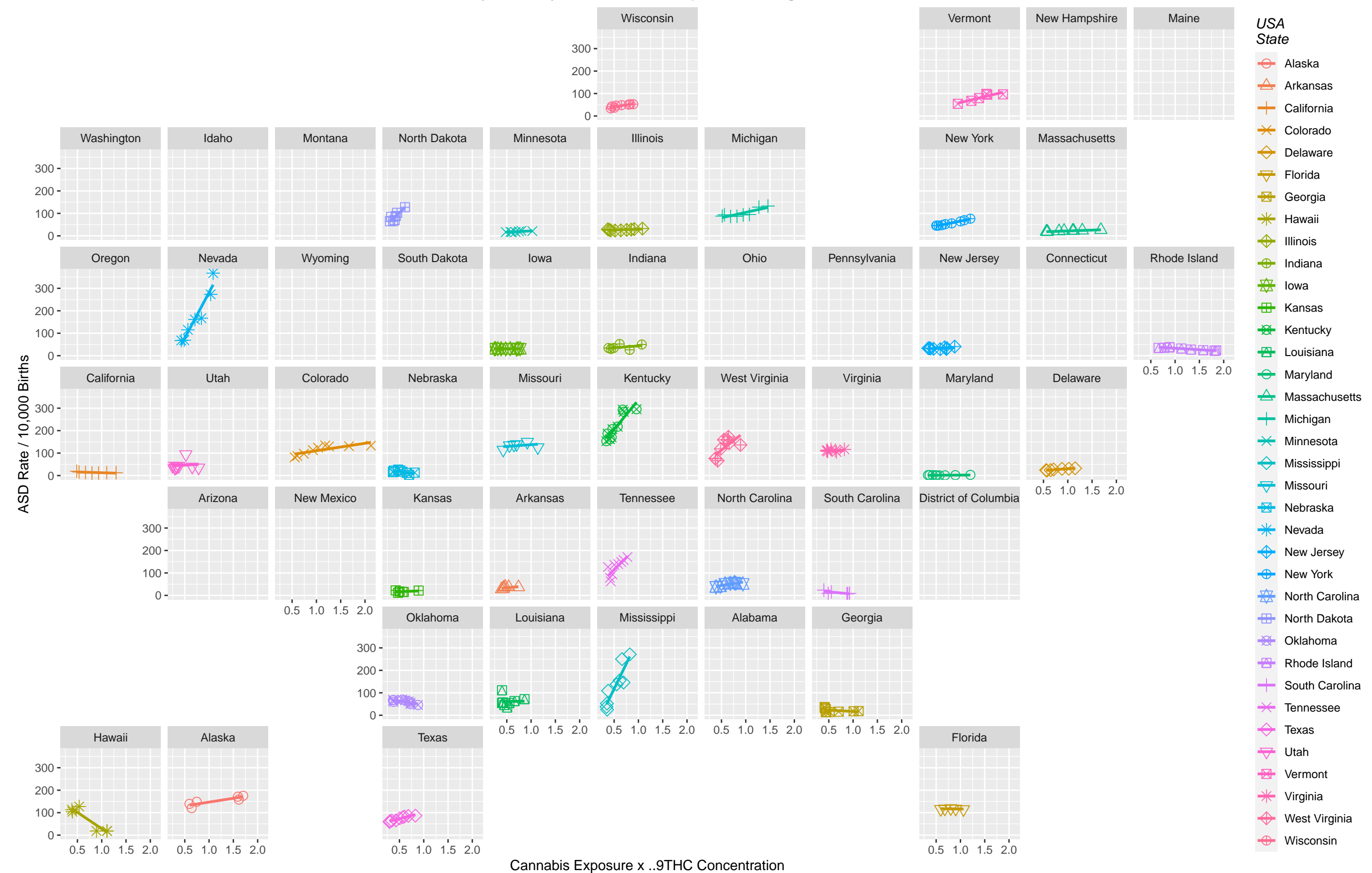

Supplement: Supplementary file 4 — Additional file 4: eFigure 3. Geofacetted plot with each US state in approximately their appropriate position, showing the ASDI over time for each state. [file 12887_2020_2431_MOESM4_ESM.pdf]

# ASD Rate by State by Cannabinoid Exposure – Regression Lines

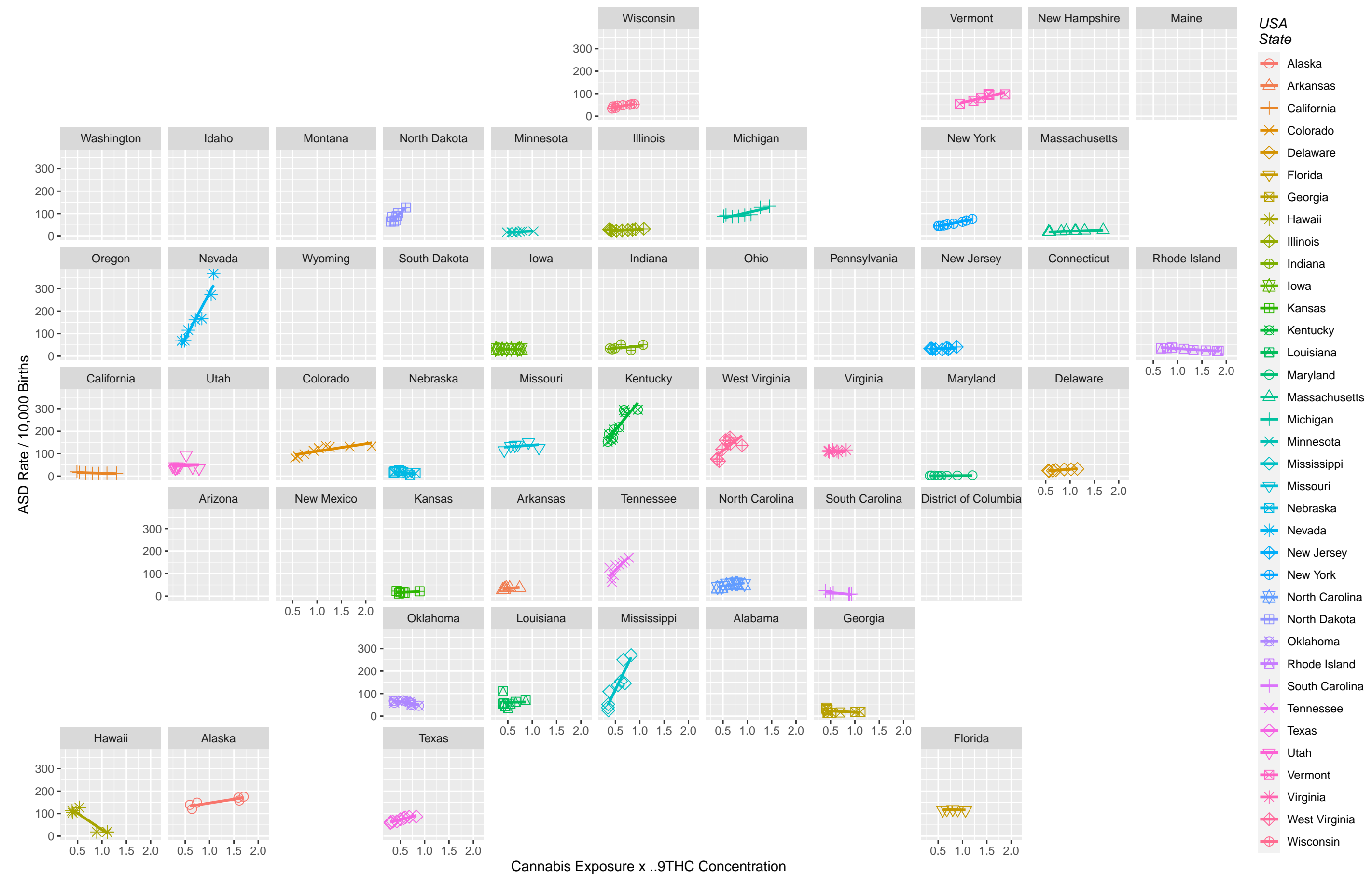

Supplement: Supplementary file 5 — Additional file 5: eFigure 4. Geofacetted plot with each US state in approximately their appropriate position, showing the ASDI over a denominator of the product of state level cannabis use by national THC concentration (which thus provides an estimate of the state level THC exposure). [file 12887_2020_2431_MOESM5_ESM.pdf]

Cannabis / Cannabinoid Exposure by Year

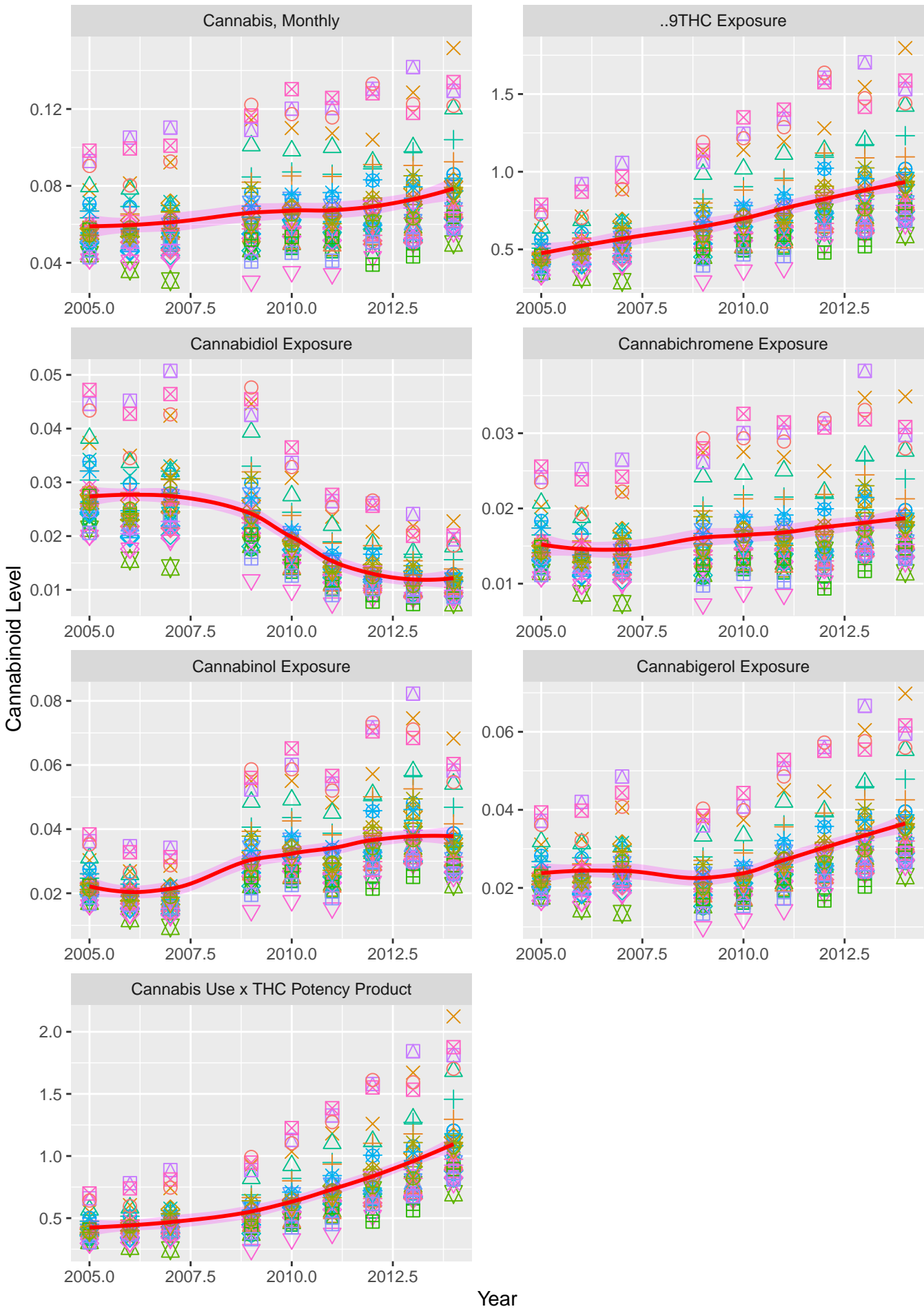

ASD Rate by Cannabidiol Exposure by Year

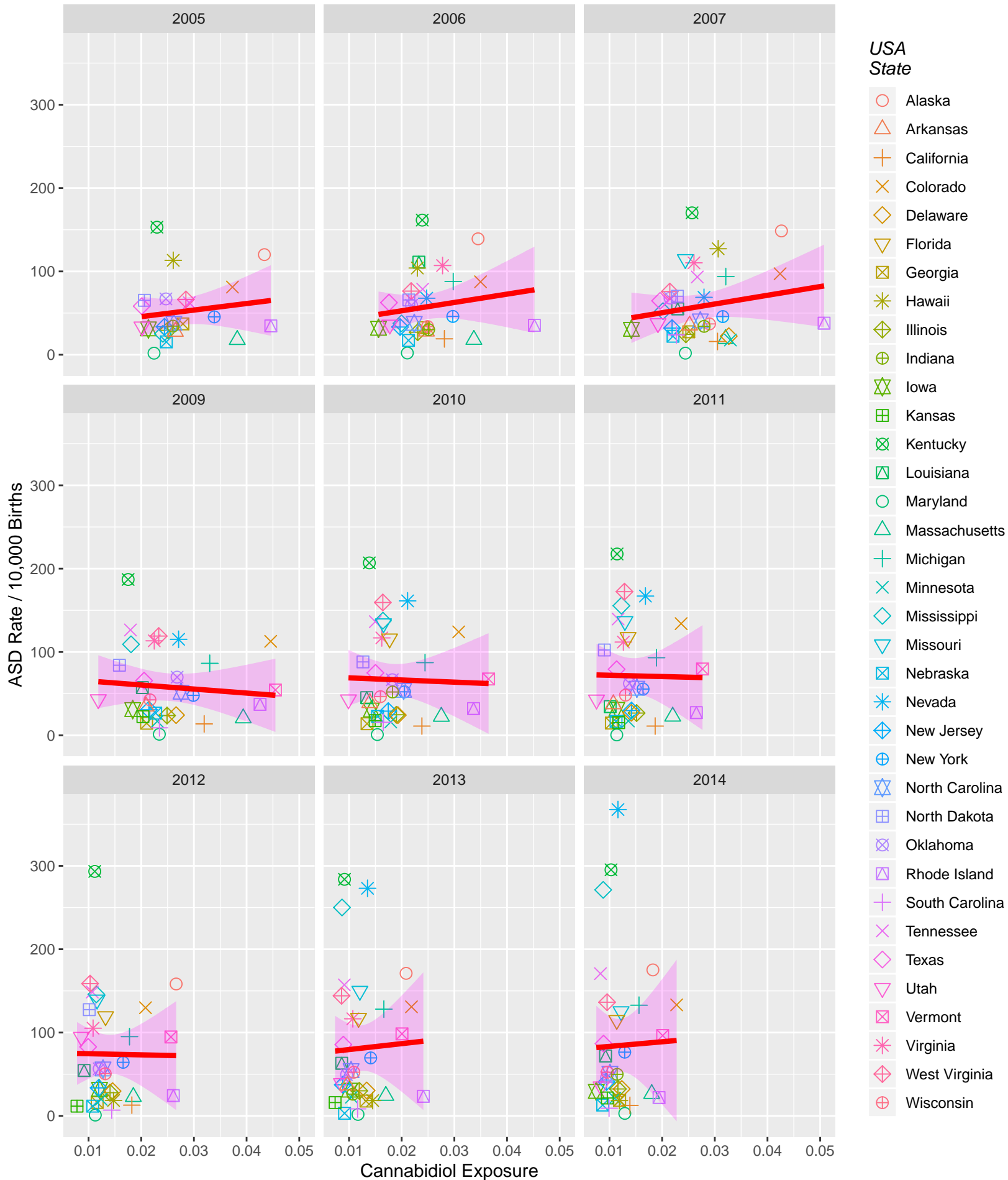

Supplement: Supplementary file 6 — Additional file 6: eFigure 5. Concentration of cannabinoids by state across time. National averaged trend line is shown in each case. (A) Selected cannabinoids with loess (localized polynomial) fitted curves. (B) Linear regression lines for state level cannabidiol exposure. [file 12887_2020_2431_MOESM6_ESM.pdf]

# ASD Rate by State by Racial Prevalence – Regression Lines

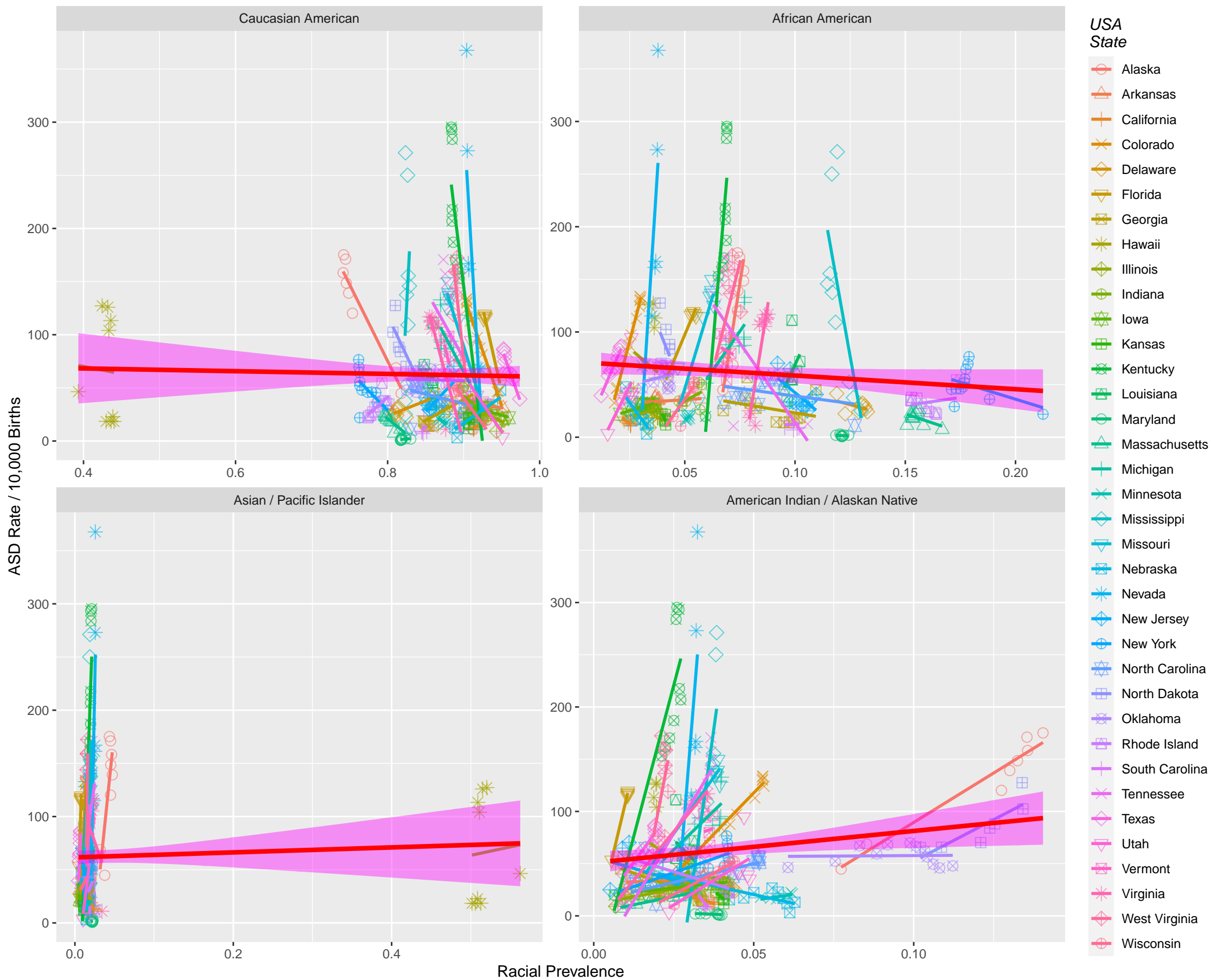

Supplement: Supplementary file 7 — Additional file 7: eFigure 6. ASD rate by ethnicity by state. [file 12887_2020_2431_MOESM7_ESM.pdf]

# ASD Rate by Median Household Income

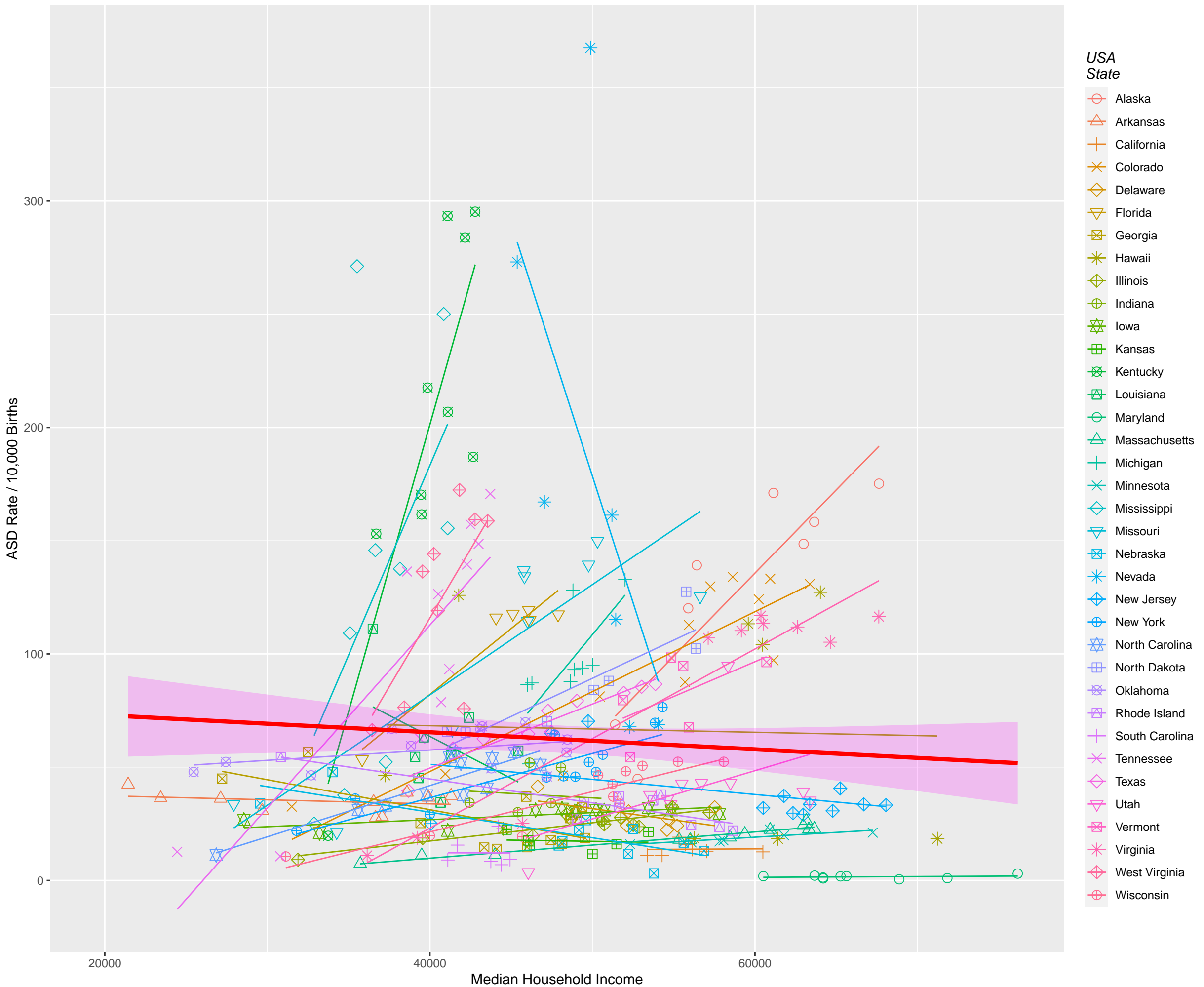

Supplement: Supplementary file 9 — Additional file 9: eFigure 8. ASD rate as a function of the median household income. [file 12887_2020_2431_MOESM9_ESM.pdf]

Log (ASD Rate) by State – Kriged Data Plot

Data: NBDPN Annual Reports

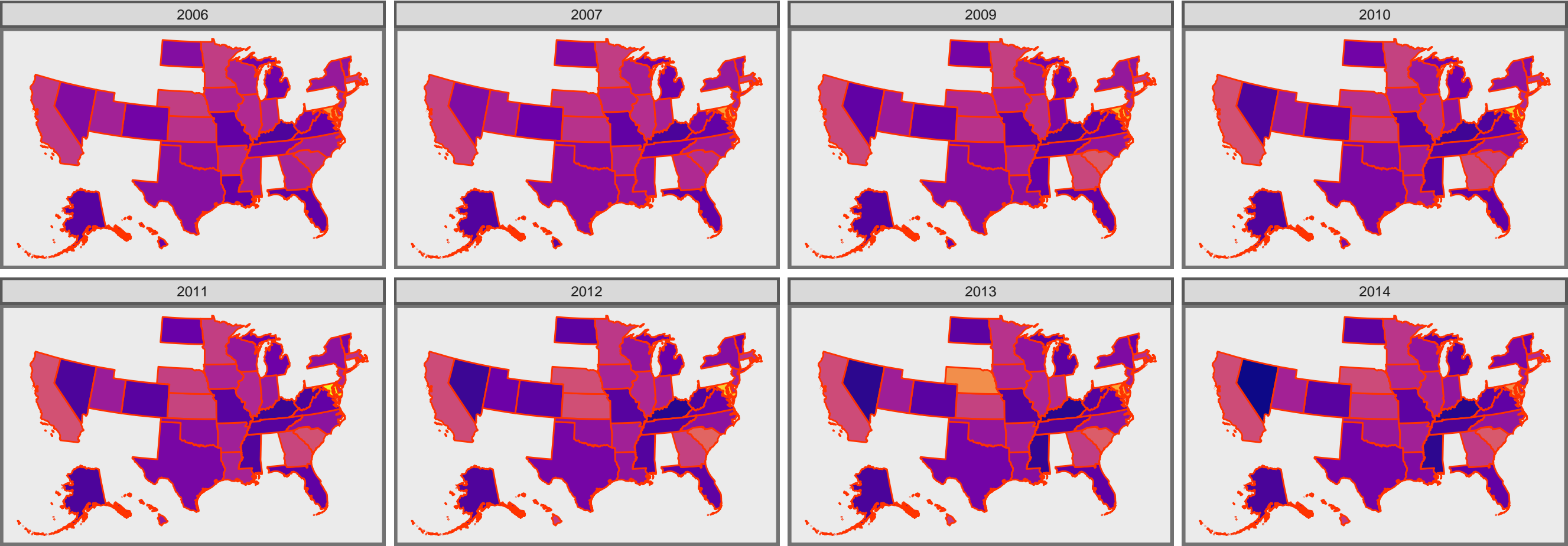

Supplement: Supplementary file 10 — Additional file 10: eFigure 9. Map-graphs depicting the log (ASDI) by state by year for the temporally kriged dataset. These maps are originals. [file 12887_2020_2431_MOESM10_ESM.pdf]
