# Supplementary material for: Contemporary epidemiology of rising atrial septal defect trends across USA 1991–2016: a combined ecological geospatiotemporal and causal inferential study
Source: BMC Pediatr. 2020 Nov 30;20:539. doi: 10.1186/s12887-020-02431-z (PMC7702707; doi:10.1186/s12887-020-02431-z)
Supplement: Supplementary file 8 — Additional file 8: eFigure 7. ASD rate by ethnicity collated for the whole nation (A) by individual ethnicity and (B) for all ethnicities pooled. [file 12887_2020_2431_MOESM8_ESM.pdf]

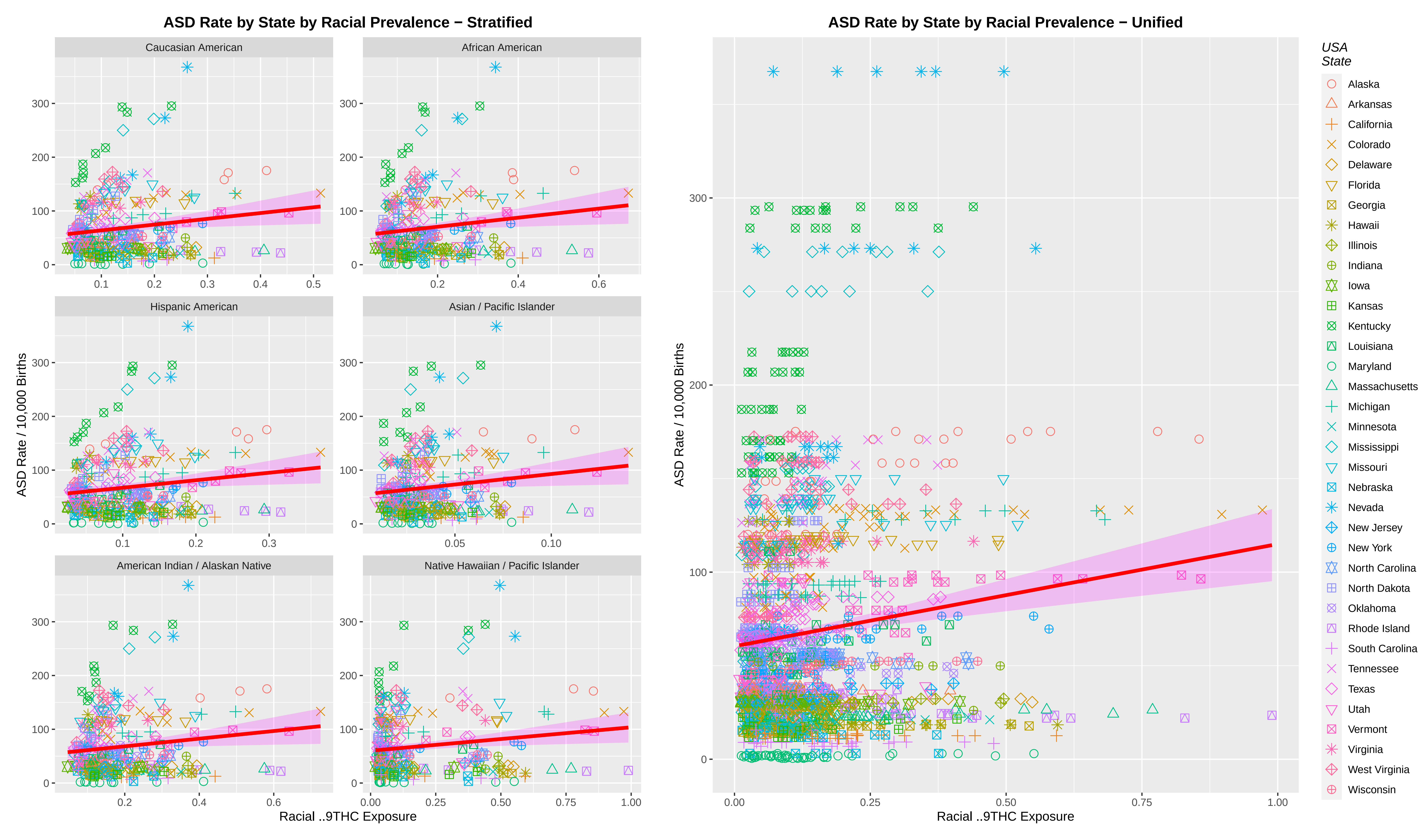

ASD Rate by State by Racial Prevalence − Stratified
Caucasian American
African American
Hispanic American
Asian / Pacific Islander
American Indian / Alaskan Native
Native Hawaiian / Pacific Islander
ASD Rate / 10,000 Births
Racial ..9THC Exposure
ASD Rate by State by Racial Prevalence − Unified
USA State
Alaska
Arkansas
California
Colorado
Delaware
Florida
Georgia
Hawaii
Illinois
Indiana
Iowa
Kansas
Kentucky
Louisiana
Maryland
Massachusetts
Michigan
Minnesota
Mississippi
Missouri
Nebraska
Nevada
New Jersey
New York
North Carolina
North Dakota
Oklahoma
Rhode Island
South Carolina
Tennessee
Texas
Utah
Vermont
Virginia
West Virginia
Wisconsin
