# Supplementary material for: Contemporary epidemiology of rising atrial septal defect trends across USA 1991–2016: a combined ecological geospatiotemporal and causal inferential study
Source: BMC Pediatr. 2020 Nov 30;20:539. doi: 10.1186/s12887-020-02431-z (PMC7702707; doi:10.1186/s12887-020-02431-z)
Supplement: Supplementary file 11 — Additional file 11: eFigure 10. Geospatial links (A) edited and (B) final used in geospatiotemporal modelling. These maps are originals. [file 12887_2020_2431_MOESM11_ESM.pdf]

A

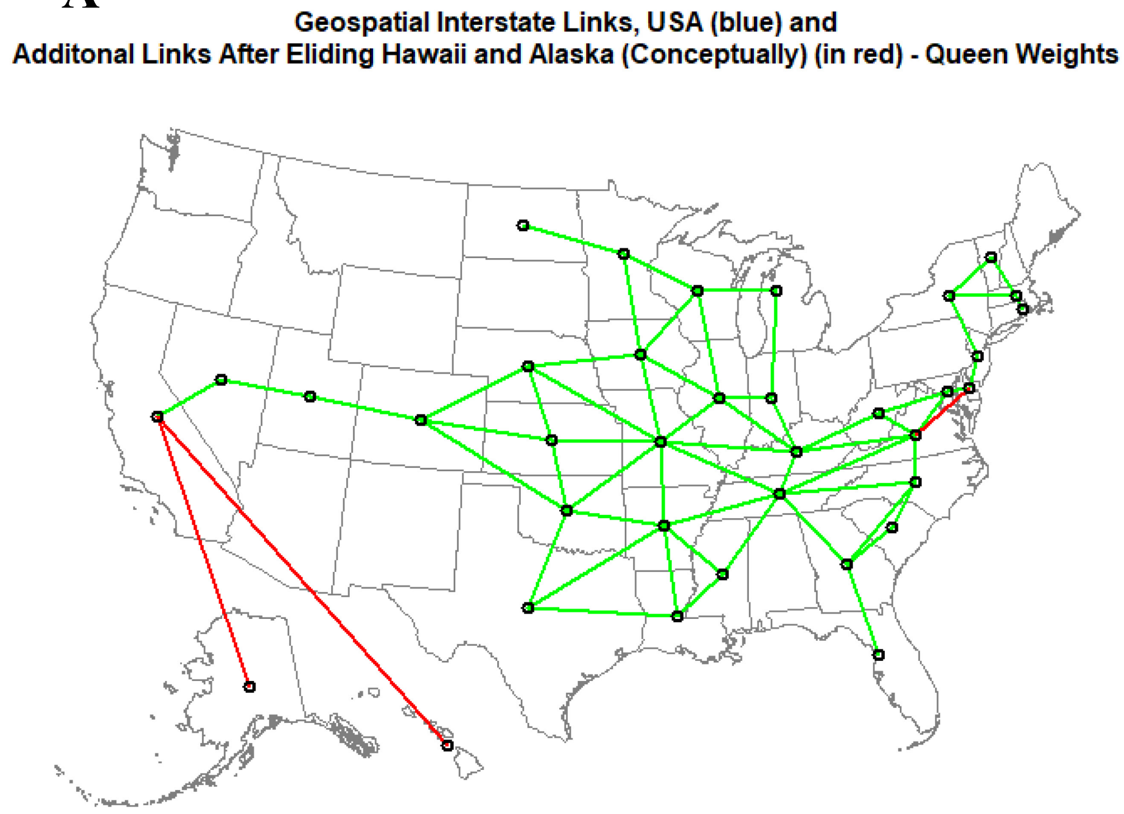
Geospatial Interstate Links, USA (blue) and
Additonal Links After Eliding Hawaii and Alaska (Conceptually) (in red) - Queen Weights

B

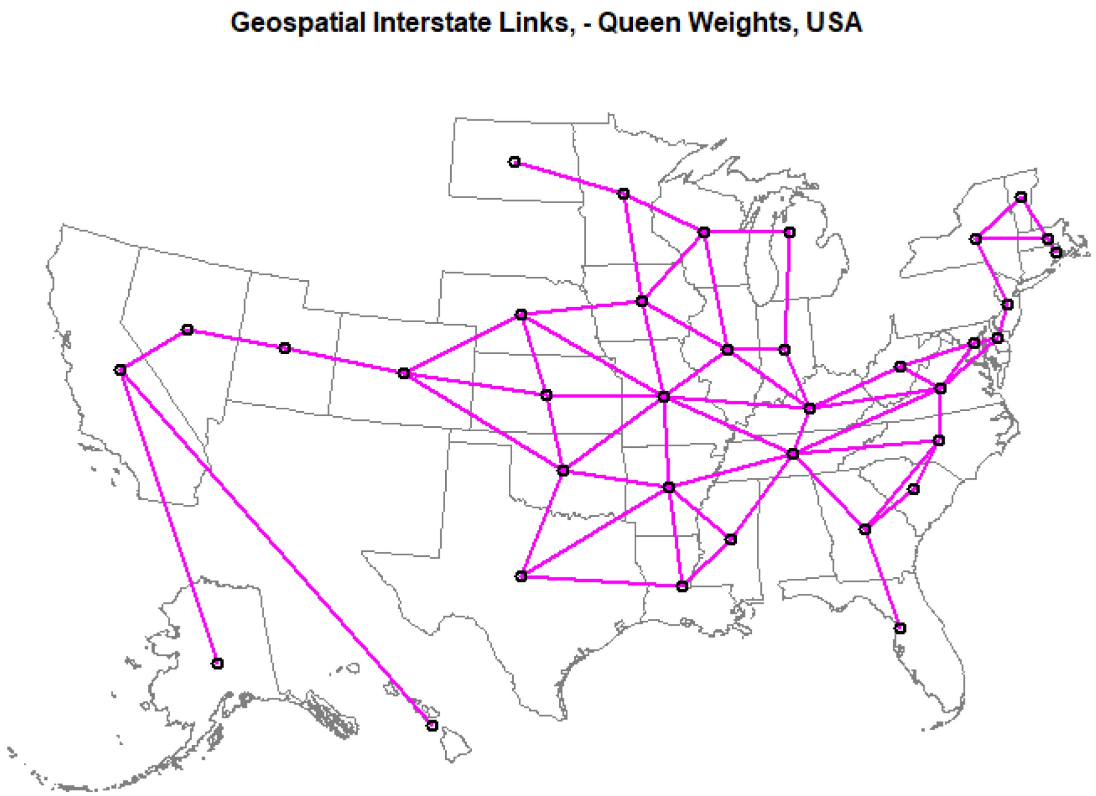
Geospatial Interstate Links, - Queen Weights, USA
